# Cytokinesis-Based Constraints on Polarized Cell Growth in Fission Yeast

**DOI:** 10.1371/journal.pgen.1003004

**Published:** 2012-10-18

**Authors:** K. Adam Bohnert, Kathleen L. Gould

**Affiliations:** Howard Hughes Medical Institute and Department of Cell and Developmental Biology, Vanderbilt University School of Medicine, Nashville, Tennessee, United States of America; University of California San Francisco, United States of America

## Abstract

The rod-shaped fission yeast *Schizosaccharomyces pombe*, which undergoes cycles of monopolar-to-bipolar tip growth, is an attractive organism for studying cell-cycle regulation of polarity establishment. While previous research has described factors mediating this process from interphase cell tips, we found that division site signaling also impacts the re-establishment of bipolar cell growth in the ensuing cell cycle. Complete loss or targeted disruption of the non-essential cytokinesis protein Fic1 at the division site, but not at interphase cell tips, resulted in many cells failing to grow at new ends created by cell division. This appeared due to faulty disassembly and abnormal persistence of the cell division machinery at new ends of *fic1*Δ cells. Moreover, additional mutants defective in the final stages of cytokinesis exhibited analogous growth polarity defects, supporting that robust completion of cell division contributes to new end-growth competency. To test this model, we genetically manipulated *S. pombe* cells to undergo new end take-off immediately after cell division. Intriguingly, such cells elongated constitutively at new ends unless cytokinesis was perturbed. Thus, cell division imposes constraints that partially override positive controls on growth. We posit that such constraints facilitate invasive fungal growth, as cytokinesis mutants displaying bipolar growth defects formed numerous pseudohyphae. Collectively, these data highlight a role for previous cell cycles in defining a cell's capacity to polarize at specific sites, and they additionally provide insight into how a unicellular yeast can transition into a quasi-multicellular state.

## Author Summary

Many processes, including cell growth, are often regulated differently in distinct cellular regions. In the rod-shaped fission yeast *Schizosaccharomyces pombe*, new cell ends created by cell division initiate growth long after old cell ends inherited from mother cells. Though distributions of cell tip factors contribute to this growth pattern, we have found that the process of cytokinesis, which executes physical separation of daughter cells at the end of the cell cycle, also plays an important role in defining new end-growth competency. Defects in completing cytokinesis and remodeling the division site curb new end growth even when protein complexes that drive tip elongation constitutively associate with new cell ends. Moreover, when parts of the cytokinetic machinery persist at the division plane following constriction, *S. pombe* cells become highly invasive. We believe that these findings provide insight into growth transitions in pathogenic fungi, as well as into the evolution of the single-celled state from multicellular hyphal forms. Additionally, we speculate that cytokinesis-based constraints on growth polarity might be conserved in mammalian cells, which have been reported to likewise polarize only distally to the cleavage furrow at the conclusion of cell division.

## Introduction

Many cells polarize in response to intrinsic and extrinsic signals. As cell polarization is generally multifaceted, cells must integrate both negative and positive cues for successful cellular morphogenesis. In various organisms, the cell cycle provides a platform on which these cues are organized (for reviews, see [1], [2]), thereby ensuring distinct polarization events occur at the appropriate location, time, and context.

The fission yeast *Schizosaccharomyces pombe* represents a genetically tractable organism for studying cell cycle regulation of growth polarity (for reviews, see [3], [4]). Wild-type *S. pombe* extend solely at their two cell tips, lengthening their rod-shaped bodies while retaining fairly constant widths. After cell division, *S. pombe* grow only at old ends, so-called because they served as ends of the dividing mother cell. Then, at a point in G2 known as new end take off (NETO), new ends, which arise from cell division, also initiate growth [5]. NETO is not required for cell viability, and myriad mutants defective in this process have been identified [3], [4]. Yet, beyond requirements for S-phase completion and a minimal interphase cell size [5], additional cell cycle controls on NETO have not been identified.

As in other cell polarization events, cytoskeletal rearrangements accompany growth transitions in *S. pombe*. Prior to NETO, microtubule plus end-associated proteins Tea1 and Tea4 ride growing microtubule ends to both cell tip cortices [6]–[9], where they anchor based on their association with membrane proteins [10], [11]. Upon NETO, Tea4 recruits formin For3, which had before only been tethered to old ends, into a complex with itself and Tea1 at new ends [8]. As over-expression of a Tea1-For3 fusion can drive NETO prematurely [8], this association likely brings For3 into the proximity of formin activators at new ends, stimulating For3 catalysis of F-actin cables that will deliver growth cargo to this tip. Not surprisingly, loss of Tea1, Tea4, and/or For3 impairs fission yeast polarization and elongation [8], [9], [12], [13]. Actin patches, which guide endocytic vesicle internalization and constitute a second F-actin structure, also re-polarize to both cell tips upon NETO [14]. Disruption of proteins comprising these structures similarly jeopardizes growth polarity establishment [15]–[17]. Thus, alteration in protein composition at cell tips is coupled tightly to cytoskeletal rearrangements.

In addition to promoting cell tip growth, several tip-localized cell polarity factors, including Tea1 and Tea4, direct the cell division plane away from cell ends and towards the cell middle for cytokinesis [18], the process by which daughter cells undergo physical separation following nuclear division. However, whether the process of cytokinesis reciprocally modulates cell polarity is unclear. Some observations hint that the cell division machinery may play a role in directing cell polarity. As was previously noted, new ends formed by cell division initiate growth well after old ends. In mutants in which cells remain physically connected at division sites for multiple cell divisions, internal cells can grow, though this occurs sub-apically adjacent to septa [19], [20]. Moreover, many polarity factors localize to the cell division site [4], [21]–[23]; nonetheless, only cell tip-localized populations of these polarity proteins have been demonstrated to contribute to growth polarity in *S. pombe*.

As in most eukaryotes, cytokinesis occurs in *S. pombe* through the assembly and constriction of an actomyosin-based cytokinetic ring (CR) [24]. In addition to actin and myosin, several accessory proteins regulate the dynamics and organization of this structure. For one, Cdc15, which contains an N-terminal F-BAR domain and a C-terminal SH3 domain characteristic of the *pombe* Cdc15 homology protein family [25], has been posited to link CR proteins to the cortical membrane at the division site [26]. Cdc15-binding proteins at the CR include formin, myosin, and the C2 domain protein Fic1 [27], [28]. Fic1 localizes to both interphase cell tips and the cell division site [28], though its specific functions at these sites have not been described. Fic1's budding yeast ortholog, Inn1, contributes to cytokinesis by linking the CR to the ingressing membrane and by participating in septum formation [29], [30]. Septa form in both budding and fission yeasts as cell wall is deposited behind the constricting CR [31]. A conserved signaling network, known as the septation initiation network (SIN) in *S. pombe*, triggers septum deposition during cytokinesis [32]. Together with the CR, septa provide mechanical force for membrane closure at the cell division site [33]. Subsequent septum degradation allows for abscission [34], [35]. Clearly, various remodeling events must occur at the cell division site for cytokinesis to complete efficiently. Whether such remodeling events also influence daughter cell behavior has never been examined.

While wild-type *S. pombe* classically grow in a single-celled form, multiple fission yeasts, including *S. pombe*, possess the ability to assume an invasive, hyphal-like state [20], [36]. The ability of pathogenic fungi to undergo such a morphogenetic switch contributes significantly to fungal infections [37]. Though non-pathogenic, *S. pombe*, similar to the budding yeast *Saccharomyces cerevisiae* [38], can transition into invasive growth as a foraging response to low nutrients [36]. Invasive *S. pombe* form structures that technically qualify as pseudohyphae, for, unlike as in hyphal growth, cytokinetic constriction occurs [39], [40]. Pseudohyphae likely maintain their hyphal-like structure due to cellular adherence and preferential growth at old ends [39], [40]. Intriguingly, it has been postulated that single-celled fission yeast evolved from multicellular, filamentous fungi, with transcriptional networks that ensure efficient cell separation playing predominant roles in the evolution of a single-celled state [41]. Though *S. pombe* pseudohyphae do not commonly exhibit aborted cytokineses or multicellularity, it is an attractive hypothesis that inefficient, but not entirely defective, cytokinesis might somehow mark new ends to impair their growth and promote the dimorphic switch in *S. pombe*.

In this manuscript, we define a novel cell cycle control on *S. pombe* growth polarity, namely that the process of cytokinesis imposes limitations on new end growth competency. Here, we focus on Fic1, which we show to be involved in the re-establishment of polarized cell growth at new ends following cell division. Specifically, we demonstrate that Fic1 polarity function requires its localization to the CR but not to interphase cell tips, and that its protein-protein interactions at the CR, including that with Cdc15, promote bipolar cell growth in the ensuing cell cycle. We present evidence that loss of Fic1 impairs disassembly of the cell division apparatus, with parts of this machinery persisting at new ends following CR constriction. Additional mutants defective in late cytokinesis also exhibit impaired new end growth. Importantly, premature activation of NETO signaling does not fully rescue bipolar growth in cells with late cytokinesis defects, suggesting that cytokinesis-based constraints on *S. pombe* growth polarity play a central role in defining new end growth competency. We propose that such constraints can provide a mechanistic understanding of how *S. pombe* and possibly other fungi transition into invasive hyphal-like growth.

## Results

### The *S. pombe* Cytokinesis Factor Fic1 Promotes the Establishment of Bipolar Cell Growth

**Figure 1:**
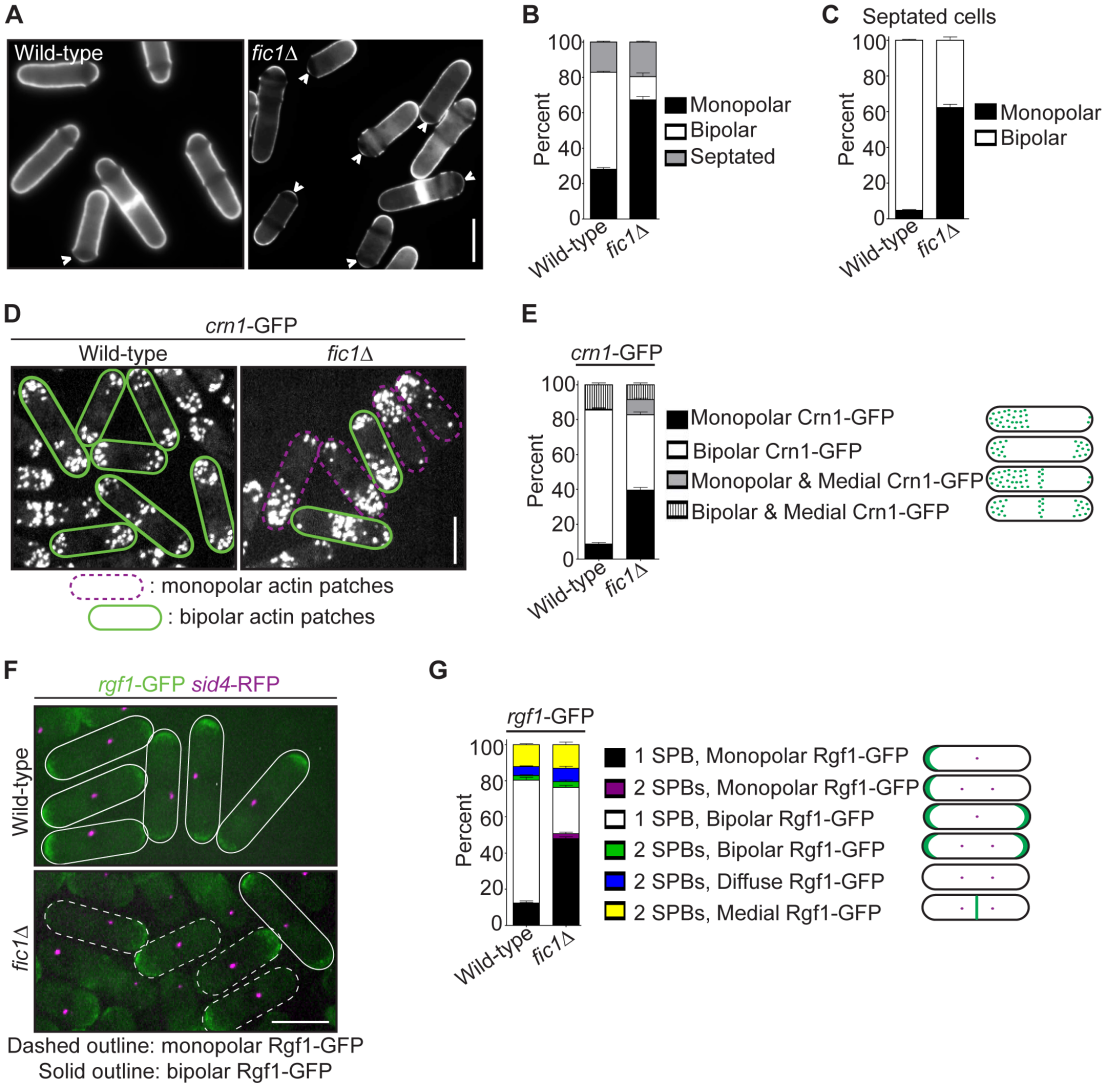
Loss of the cytokinesis protein Fic1 causes defects in *S.* *pombe* growth polarity. (A) Live-cell images of calcofluor-stained wild-type and *fic1*Δ cells. Birth scars remain unstained and appear as dark bands across cells. Arrowheads indicate monopolar cells, i.e. cells that have only grown at one end, with birth scars abutting cell ends. (B) Quantification of (A), with three trials per genotype and n>300 for each trial. Data are presented as mean ± SEM for each category. (C) Quantification of septated cells in (A) and (B), with three trials per genotype and n>200 for each trial. Data are presented as mean ± SEM for each category. (D) Live-cell GFP images of *crn1*-GFP and *fic1*Δ *crn1-*GFP cells. (E) Quantification of (D), with three trials per genotype and n>200 for each trial. Data are presented as mean ± SEM for each category. (F) Live-cell GFP (in green) and RFP (in magenta) merged images of *rgf1*-GFP *sid4*-RFP and *fic1Δ rgf1*-GFP *sid4*-RFP cells. (G) Quantification of (F), with three trials per genotype and n>200 for each trial. Data are presented as mean ± SEM for each category (Bars = 5 µm).

Recently, our laboratory identified Fic1, which was implicated in cytokinesis based on its protein and genetic interactions and its localization to the CR [28]. In addition to defects in cytokinesis, deletion of *S. pombe fic1^+^*, which is a non-essential gene, resulted in an abnormally high percentage of cells that grew only from one end (i.e., monopolar cells) (Figure 1A–1C). Tip growth was judged using calcofluor staining, as birth scars formed at previous division sites do not stain well with calcofluor and growth can be assessed using the position of these scars relative to cell tips (Figure S1A) [5]. The growth defects observed upon *fic1^+^* disruption suggested that Fic1 not only participates in cytokinesis but also in the establishment of bipolar cell growth.

Although the upstream NETO factors Tea1 and Tea4 localized normally to both cell tips in *fic1*Δ cells (Figure S1B–S1C), other cell tip proteins implicated in growth polarity regulation exhibited unusual localization patterns in this mutant. Unlike wild-type cells with mostly bipolar actin patch distribution (Figure 1D–1E), a variety of mutants defective in bipolar cell growth exhibit monopolar actin patches [8], [21]–[23]. As in such mutants, the actin patch marker Crn1-GFP [42] accumulated preferentially at one cell end in a high percentage of *fic1*Δ cells (Figure 1D–1E). Signaling through Rho GTPases controls actin patch organization in *S. pombe* [13], [43], and the Rho1 activator Rgf1 [21], which was GFP-tagged and imaged with the spindle pole body marker Sid4-RFP [44], likewise predominated on one end of many *fic1*Δ cells (Figure 1F–1G). Not surprisingly, in both wild-type and *fic1*Δ cells, Rgf1-GFP and Crn1-RFP concentrated at the same ends (Figure S1D), which were confirmed by calcofluor staining to be the growing ends of *fic1*Δ cells (Figure S1E). Consistent with Fic1 affecting both actin and Rho networks, deletion of *fic1^+^* was synthetically sick with deletion of genes encoding factors involved in F-actin nucleation (WASp Wsp1) and Rho GTPase regulation (RhoGEF Rgf1 and RhoGAP Rga1) (Figure S1F). Thus, we conclude that the absence of Fic1 upsets patterning of some but not all polarity factors.

**Figure 2:**
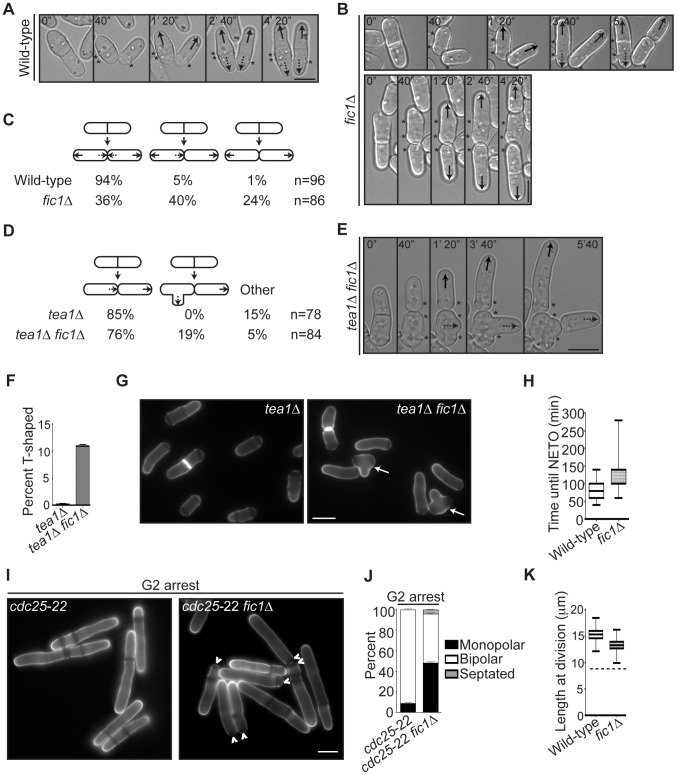
*fic1*Δ cells fail at new end growth independently of known cell cycle controls on NETO. (A–B) Live-cell DIC movies of wild-type or *fic1*Δ cells. Solid arrows denote old end growth, whereas dashed arrows indicate new end growth. Birth scars are marked by asterisks. Time points are noted. (C) Quantification of growth patterns for cells imaged in (A) and (B), with sample size (n) provided. (D) Quantification of growth patterns for *tea1*Δ and *tea1*Δ *fic1*Δ cells, with sample size (n) provided. (E) Live-cell DIC movie of a *tea1*Δ *fic1*Δ cell that gives rise to a T-shaped daughter cell. The solid arrow denotes old end growth, whereas the dashed arrow indicates non-tip growth. Birth scars are marked by asterisks. Time points are noted. (F) Quantification of T-shaped cells in *tea1*Δ and *tea1*Δ *fic1*Δ strains grown at 25°C, with three trials per genotype and n>300 for each trial. Data are presented as mean ± SEM for each genotype. (G) Live-cell images of calcofluor-stained *tea1*Δ and *tea1*Δ *fic1*Δ cells grown at 25°C. Arrows indicate T-shaped cells. (H) Quantification of times from septum splitting to initiation of new end growth in cells that undergo NETO prior to the next septation in (A–C). Data are presented in box-and-whisker plots showing the median (line in the box), 25^th^–75^th^ percentiles (box), and 5^th^–95^th^ percentiles (whiskers) for each genotype. (I) Live-cell images of calcofluor-stained *cdc25*-22 and *fic1*Δ *cdc25-*22 cells that had been arrested in G2. Arrowheads indicate monopolar cells. (J) Quantification of (I), with three trials per genotype and n>300 for each trial. Data are presented as mean ± SEM for each category. (K) Quantification of cell lengths at cell division, with n>200 for each genotype. Data are presented as box-and-whisker plots showing the median (line in the box), 25^th^–75^th^ percentiles (box), and 5^th^–95^th^ percentiles (whiskers) for each genotype. The dashed line represents the minimum length required for NETO [5] (Bars = 5 µm).

To discern whether new and/or old ends were defective in resuming growth following cell division in *fic1*Δ cells, we performed time-lapse DIC imaging to trace birth scars in live cells. As expected, nearly all wild-type cells underwent NETO prior to subsequent septation (Figure 2A and 2C). However, following roughly two-thirds of *fic1*Δ cell divisions, either one or both daughter cells failed to initiate new end growth prior to the next septation (Figure 2B–2C). The most predominant growth pattern in *fic1*Δ cells was that in which one daughter cell underwent NETO while the other did not (Figure 2B–2C), with nearly 70% of those daughter cells that did not exhibit NETO being the younger daughter cell. Unlike *tea1*Δ and *tea4*Δ cells, in which one daughter cell commonly fails at its new end and the other daughter cell fails at its old end (Figure 2D) [8], [22], [23], *fic1*Δ cells were specifically defective in the re-establishment of growth at new ends following cell division (Figure 2B–2C). Intriguingly, *tea1*Δ *fic1*Δ double mutants grew mainly in a *tea1*Δ pattern, though nearly one-fifth of cell divisions produced a T-shaped daughter cell (Figure 2D–2E). Consistent with this, roughly 10% of *tea1*Δ *fic1*Δ cells were T-shaped at 25°C, while T-shaped *tea1*Δ cells were almost never observed at this temperature (Figure 2F). T-shapes always arose in cells that the *tea1*Δ growth pattern dictated should grow at their new ends (Figure 2D–2E) but that actually grew at neither (Figure 2E and 2G), suggesting these cells polarize at sites other than their tips because growth is inhibited at both ends. These data confirmed that the polarity defect caused by loss of Fic1 stochastically impacts new end growth in a variety of genetic backgrounds. Importantly, *fic1*Δ new ends that failed to extend in one cell cycle initiated growth as an old end in the next cell cycle, suggesting the defect in growth polarity caused by loss of Fic1 was not permanent. Consistent with a delay but not a block in growth, new ends that initiated growth prior to the next septation did so much later on average in *fic1*Δ cells than in wild-type cells (120 min versus 75 min) (Figure 2H).

To test whether *fic1*Δ's polarity defect was independent of S phase completion, we arrested *fic1*Δ cells in late G2 using *cdc25-*22, a temperature-sensitive allele of the phosphatase that activates cyclin-dependent kinase at the G2-M transition. As was previously observed [5], otherwise wild-type cells blocked in G2 almost always underwent NETO (Figure 2I–2J). However, roughly half of *fic1*Δ cells remained monopolar (Figure 2I–2J), indicating that the *fic1*Δ polarity defect occurs irrespective of S phase completion. To test whether *fic1*Δ cells were too small to initiate NETO, we measured cell lengths at division. Though slightly shorter on average than wild-type cells (13.3 µm versus 15.3 µm), all *fic1*Δ cells were longer at division than the minimum length required for NETO (∼9 µm) (Figure 2K) [5]. Therefore, it is unlikely that the *fic1*Δ growth polarity defect is caused by reduced cell length. These data underscore that loss of Fic1 disrupts the establishment and timing of NETO independently of previously described cell cycle controls.

### Fic1 Protein–Protein Interactions at the CR Support Subsequent Polarized Cell Growth at New Ends

**Figure 3:**
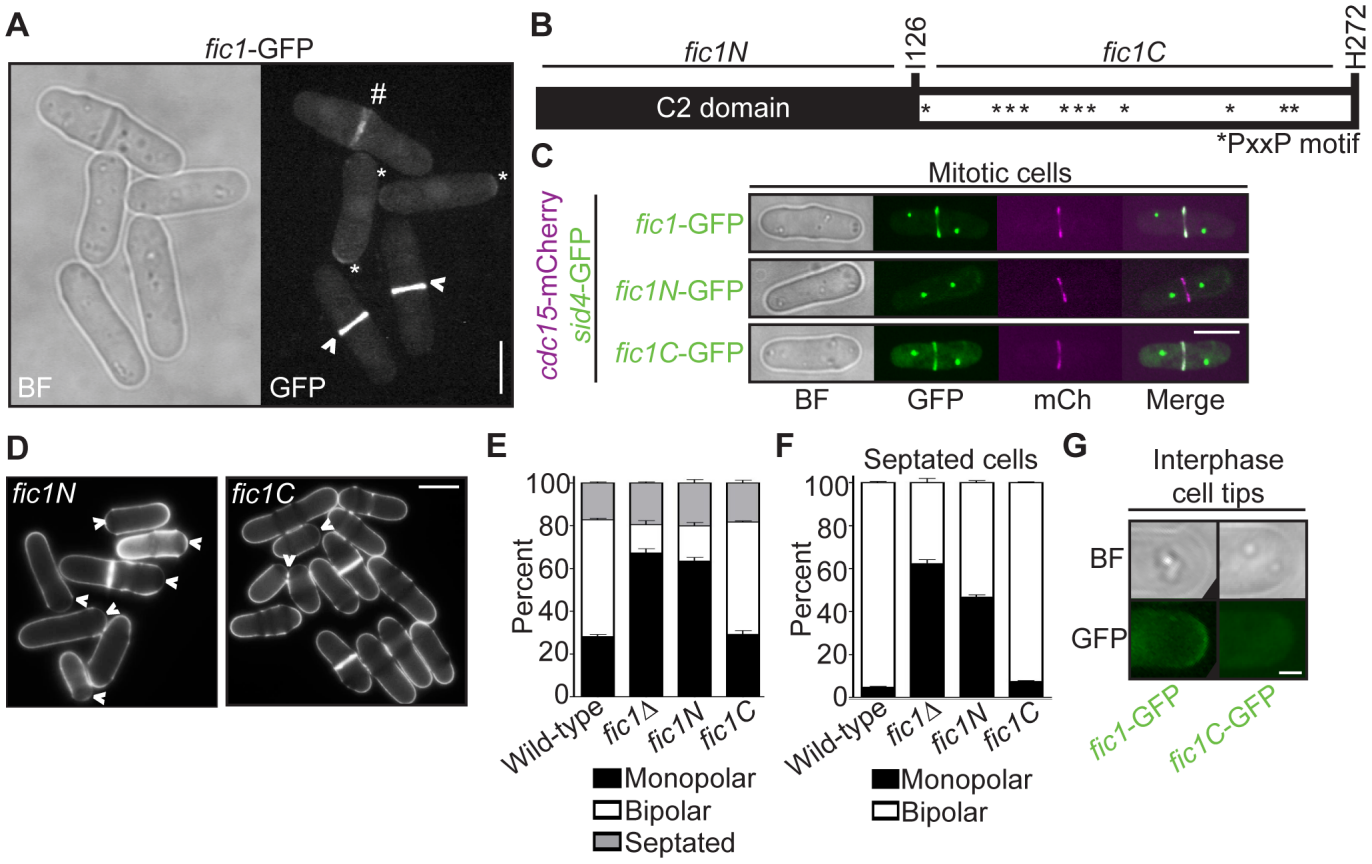
Fic1's C terminus is necessary and sufficient for Fic1's polarity function at the division site. (A) Live-cell bright field (BF) and GFP images of *fic1*-GFP cells. Localization to cell tips (*), the cytokinetic ring (>), and the division site (#) are marked. (B) Schematic of Fic1 protein domain organization. Residues and fragments of interest are marked. (C) Live-cell BF, GFP (in green), mCherry (mCh) (in magenta), and GFP/mCherry merged images of *fic1*-GFP *sid4*-GFP *cdc15*-mCherry, *fic1N*-GFP *sid4*-GFP *cdc15*-mCherry, and *fic1C*-GFP *sid4*-GFP *cdc15*-mCherry cells. (D) Live-cell images of calcofluor-stained *fic1N* and *fic1C* cells. Arrowheads indicate monopolar cells. (E) Quantification of (D), with three trials per genotype and n>300 for each trial. Data are presented as mean ± SEM for each category. (F) Quantification of septated cells in (D) and (E), with three trials per genotype and n>200 for each trial. Data are presented as mean ± SEM for each category. (G) Live-cell BF and GFP images of interphase cell tips of *fic1*-GFP and *fic1C-*GFP cells (Bars = 5 µm, except for 3G where Bar = 1 µm).

Though many cell polarity factors localize to the cell division site in addition to interphase cell tips, only the actions of these proteins at interphase cell tips have been demonstrated to be relevant to polarity regulation. As was observed previously [28], cytoplasmic Fic1-GFP localizes to cell tips during interphase and later to the CR during cell division (Figure 3A). We also detected another pool of Fic1-GFP lining the division site as the CR constricted (Figure 3A). Given this localization pattern and the specific new end growth defect of *fic1*Δ cells, we asked whether Fic1 affected the timing of NETO via its functions at the cell division site.

Like *S. cerevisiae* Inn1 [30], Fic1 is comprised of an N-terminal C2 domain and a C-terminal stretch of PxxP motifs (Figure 3B). As was found for Inn1 [29], [30], the C terminus of Fic1 (“Fic1C”, amino acids 127–272), expressed from its endogenous locus and GFP-tagged, was sufficient for CR localization, as judged by co-localization with the CR protein Cdc15-mCherry (Figure 3C). In contrast, a GFP-tagged N-terminal C2 domain fragment (“Fic1N”, amino acids 1–126) was never observed at the CR (Figure 3C) though it was produced *in vivo* (Figure S2). Importantly, medial-localizing Fic1C, unlike Fic1N, supported proper growth polarity establishment (Figure 3D–3F), and, in contrast to full-length Fic1-GFP, Fic1C-GFP was not detected at tips of interphase cells (Figure 3G). We thus conclude that Fic1, unlike other characterized growth polarity factors, does not exert its polarity function at cell tips during interphase, but instead does so at the cell division site during cytokinesis.

**Figure 4:**
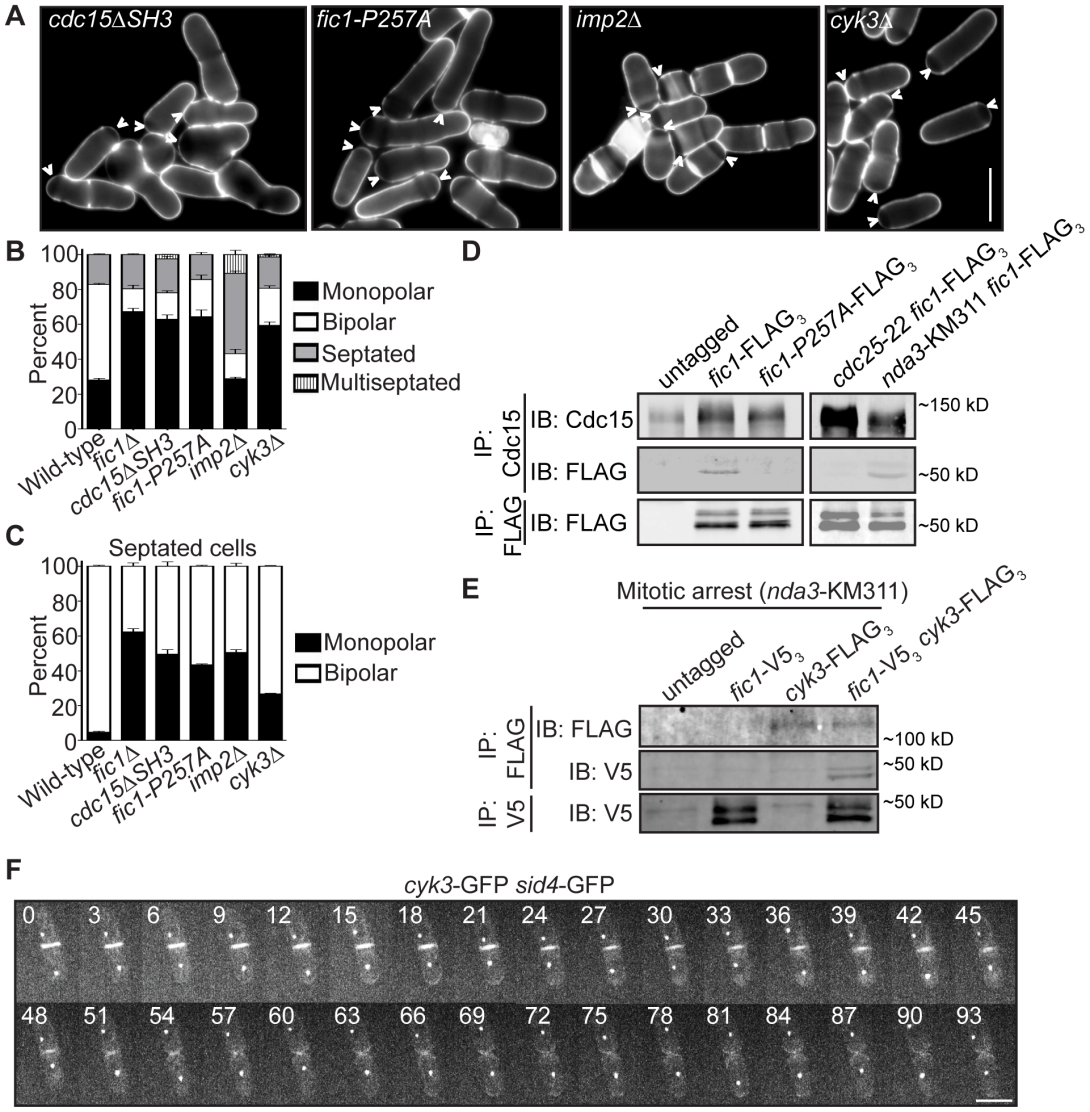
Fic1 participates in protein–protein interactions at the CR that guide subsequent growth polarity. (A) Live-cell images of calcofluor-stained *cdc15*Δ*SH3*, *fic1-P257A*, *imp2*Δ, and *cyk3*Δ cells. Arrowheads indicate monopolar cells. (B) Quantification of (A), with three trials per genotype and n>300 for each trial. Data are presented as mean ± SEM for each category. (C) Quantification of septated cells in (A) and (B), with three trials per genotype and n>300 for each trial. Data are presented as mean ± SEM for each category. (D) Anti-Cdc15 and anti-FLAG immunoprecipitates from cells of the indicated genotypes were blotted with anti-Cdc15 and/or anti-FLAG antibodies. *cdc25*-22 cells and *nda3*-KM311 cells were arrested in G2 and prometaphase, respectively, prior to pelleting and lysis. (E) Anti-FLAG and anti-V5 immunoprecipitates from prometaphase-arrested cells of the indicated genotypes were blotted with anti-FLAG and/or anti-V5 antibodies. (F) Live-cell GFP movie of a *cyk3*-GFP *sid4*-GFP cell, with images every 3 min (Bars = 5 µm).

Because Fic1's C terminus was necessary and sufficient for proper growth polarity, we examined whether protein-protein interactions at the CR mediated by Fic1's C-terminal PxxP motifs, which bind SH3 domains, govern Fic1's polarity function. Fic1 was originally identified based on its interaction with Cdc15's SH3 domain [28]. As would be expected if association of Cdc15 with Fic1's C terminus is important in establishing the timing of NETO, calcofluor-stained *cdc15*Δ*SH3* cells, which are viable but lack Fic1-Cdc15 interaction [28], exhibited growth polarity defects (Figure 4A–4C and Figure S3A–S3B).

To address the consequence of specifically disrupting Fic1-Cdc15 interaction, we determined which of Fic1's C-terminal PxxP motifs interact(s) with Cdc15's SH3 domain. Previous yeast-two hybrid data indicated Fic1 amino acids 190–269 mediate direct association with Cdc15's SH3 domain [28]. This region contains four of the eleven PxxP motifs within Fic1's C terminus (Figure S3C). To identify which are relevant for Cdc15 interaction, yeast two-hybrid assays using single and combinations of proline to alanine mutations were performed (Figure S3D). Mutation of PxxPs 10 and 11 in combination, or P257 of PxxP 11 alone, abolished the two-hybrid interaction (Figure S3D), and the P257A mutation eliminated co-immunoprecipitation of Fic1-FLAG_3_ with Cdc15 *in vivo* (Figure 4D). Supporting the idea that the Fic1-Cdc15 interaction is most relevant during cell division, Fic1-GFP did not accumulate preferentially in Cdc15-mCherry puncta at interphase cell tips (Figure S3E) and co-immunoprecipitation of Fic1-FLAG_3_ with Cdc15 was considerably stronger in mitosis than in interphase (Figure 4D). This is similar to other Cdc15 protein-protein interactions, which become enriched upon Cdc15 dephosphorylation at mitosis [26]. *fic1-P257A* cells exhibited monopolar growth defects similar to *fic1*Δ and *cdc15*Δ*SH3* cells (Figure 4A–4C), confirming that binding of Fic1's C terminus to Cdc15 is critical for Fic1's polarity function. Even so, Fic1-P257A-GFP still localized to the CR (Figure S3F), indicating that medial localization of Fic1 during cytokinesis is necessary but not sufficient for re-establishment of proper growth polarity following cell division.

To corroborate that PxxP-mediated protein-protein interactions at the cytokinetic ring play a predominant role in Fic1's polarity function, we tested whether other interactors participate in *S. pombe* polarity regulation. The SH3 protein Imp2 has previously been shown to function redundantly with Cdc15 and bind Fic1 during cytokinesis [28]. Consistent with additional Fic1 interactions guiding growth polarity, loss of Imp2 also severely compromised bipolar cell growth (Figure 4A–4C and Figure S3A–S3B). In *S. cerevisiae*, the Fic1 ortholog Inn1 binds to another SH3 protein, Cyk3 [29], [45], and complexing of these two proteins with the Cdc15 homolog Hof1 has been suggested to direct septum formation and cell separation [29]. We found that *S. pombe* Cyk3 co-immunoprecipitated with Fic1 in mitosis (Figure 4E), and we also detected direct interaction between S. *pombe* Cyk3's SH3 domain and Fic1 via yeast two-hybrid (Figure S3G). Accordingly, these interactions appear to be conserved. As was also described in a recent study [46], we found that Cyk3-GFP localized to the CR and division site during cytokinesis, and it was retained at new ends immediately following cell division (Figure 4F). Consistent with these proteins performing a common function, loss of Cyk3 resulted in growth polarity defects similar to those seen upon loss of Fic1 or its interaction with Cdc15 or Imp2 (Figure 4A–4C). Thus, Fic1 collaborates with associated proteins at the CR to execute its growth polarity function, and we postulate that its C terminus acts as an adaptor molecule for SH3 proteins to ensure integration of distinct processes during cytokinesis. Of note, Fic1-P257A-GFP still localized to the CR in *imp2*Δ *cyk3*Δ cells (Figure S3H), indicating other CR proteins besides Cdc15, Imp2, and Cyk3 bind Fic1 and likely participate in polarity-relevant events at the division site.

### Loss of Fic1 Impedes CR Disassembly and Leads to Persistence of Factors at the Division Site

**Figure 5:**
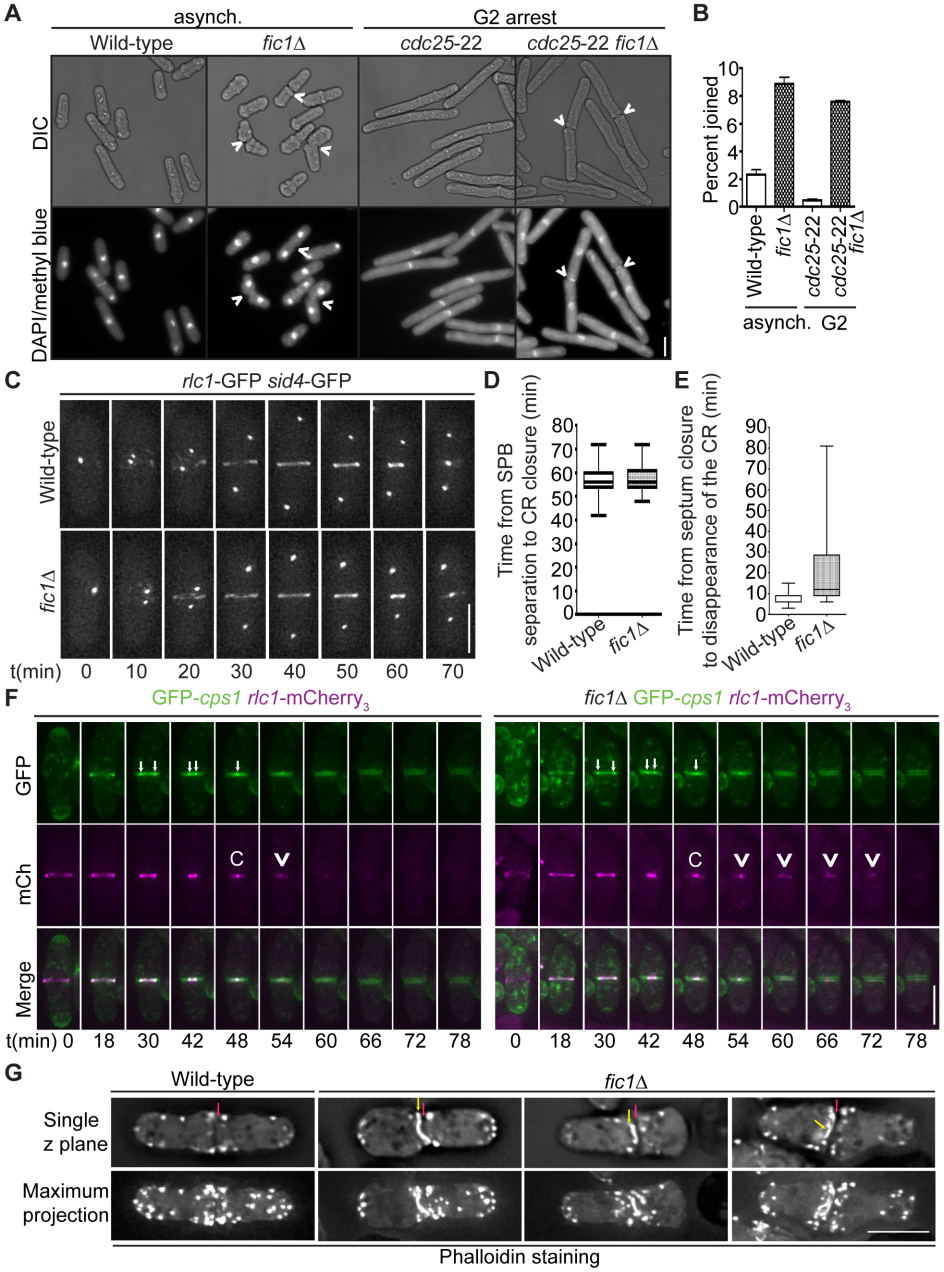
Loss of Fic1 impairs CR disassembly and leads to persistence of division site factors. (A) Fixed-cell DIC and DAPI/methyl blue images of asynchronous and G2-arrested cells of the indicated genotypes. Arrowheads indicate cells that are still joined following ingression. (B) Quantification of (A), with four trials per genotype and n>300 for each trial. Percentages are presented as mean ± SEM. (C) Live-cell GFP movies of *rlc1*-GFP *sid4*-GFP and *fic1*Δ *rlc1*-GFP *sid4*-GFP cells. Images were acquired every 2 min, and representative images are given for 10 min intervals. (D) Quantification of times from spindle pole body (SPB) separation to the completion of CR constriction in (C). n>20 for each genotype. Data are presented in box-and-whisker plots showing the median (line in the box), 25^th^–75^th^ percentiles (box), and 5^th^–95^th^ percentiles (whiskers) for each genotype. (E) Quantification of times from septum closure to disappearance of the CR at the division site for GFP*-cps1 rlc1-*mCherry_3_ and *fic1*Δ GFP*-cps1 rlc1-*mCherry_3_ cells. n>30 for each genotype. Data are presented in box-and-whisker plots showing the median (line in the box), 25^th^–75^th^ percentiles (box), and 5^th^–95^th^ percentiles (whiskers) for each genotype. (F) Live-cell GFP (colored green) and mCherry (mCh) (colored magenta) movies of GFP*-cps1 rlc1-*mCherry_3_ and *fic1*Δ GFP*-cps1 rlc1-*mCherry_3_ cells, with time intervals indicated and GFP/mCherry images merged. White arrows in GFP images mark the septa's leading edges. The time point with only one arrow drawn marks septum closure. In the mCh images, “C” marks the point of CR closure, and arrowheads denote CR remnants persisting after this point. (G) Fixed-cell images of actin stained with Alexa Fluor 488 Phalloidin. Single z planes as well as maximum projections of multiple z planes are given. Red arrows indicate division planes, whereas yellow arrows indicate unusual actin masses lining the division plane (Bars = 5 µm).

To discern how loss of Fic1 scaffold function during cytokinesis impacts subsequent new end growth, we next defined what aspects of cytokinesis are perturbed in *fic1*Δ cells. Previous data demonstrated that *fic1*Δ was synthetically lethal with *sid2*-250 [28], a temperature-sensitive allele of the SIN kinase Sid2. Consistent with Fic1 and associated factors working in parallel to the SIN, we found that *fic1*Δ and *cyk3*Δ suppressed the hyperactive SIN allele *cdc16*-116 (Figure S4A), and that *fic1*Δ and *cyk3*Δ were synthetically sick or lethal with a variety of SIN alleles conferring loss of SIN function (Figure S4A–S4B). These genetic data implied that Fic1 most likely functions during late stages of cytokinesis. In line with this idea, the percentage of *fic1*Δ cells that had undergone ingression but were still joined at their division sites was more than four times that of wild-type cells (Figure 5A–5B). When cells were arrested in G2 using the *cdc25-*22 allele, this difference increased, with the percentage of joined cells roughly 15 times greater in the absence of Fic1 (Figure 5A–5B). Similar to *S. cerevisiae inn1*Δ cells [29] and *S. pombe cdc15*Δ*SH3* cells [28], many G2-arrested *fic1*Δ daughter cells that were still joined at division sites exhibited membranous bridges (Figure S4C). These findings verified that the completion of cell division is perturbed in the absence of Fic1.

Consistent with early cytokinesis events proceeding appropriately without Fic1, time-lapse imaging of myosin regulatory light chain Rlc1-GFP [47], [48] along with spindle pole body marker Sid4-GFP revealed that the CR formed and constricted normally in *fic1*Δ cells (Figure 5C–5D). However, at the termination of CR constriction, parts of the CR persisted at the division plane (Figure 5E–5G and Figure S4D). During cytokinesis, the septum closes behind the constricting CR, and septum closure can be visualized using the β-glucan synthase GFP-Cps1 [49], [50]. As cytokinesis progresses, two GFP-Cps1 dots marking the leading edge of the septum can be seen getting progressively closer in the division plane, and these dots eventually join into one just as the CR completes constriction (Figure 5F). We found that Rlc1-mCherry_3_ remained at the division site following septum closure on average longer in *fic1*Δ cells compared to wild-type cells (22 min versus 8 min) (Figure 5E–5F). Consistent with these remnants representing the CR as a whole and not just Rlc1, phalloidin staining revealed atypical actin-rich masses, in addition to normal actin patches, flanking septa in *fic1*Δ cells (Figure 5G). By expressing LifeAct-GFP, we verified that these abnormal actin masses co-localized to a high degree with Rlc1-mCherry_3_ in a *fic1*Δ genetic background (Figure S4D). Thus, we conclude that the CR does not disassemble properly at the conclusion of cell division in *fic1*Δ cells.

In addition to CR-associated factors, glucanase Eng1-GFP [35] persisted at ingressed division sites significantly longer in *fic1*Δ cells compared to wild-type cells (on average, 51 min versus 21 min) (Figure S4E–S4F). Because glucanases execute septum degradation [34], [35], these data suggest that cell wall turnover is inefficient at *fic1*Δ septa. We thus conclude that loss of Fic1 jeopardized the completion of cell division, stalling remodeling of new ends in the next cell cycle.

### Mutants with Late Cytokinesis Defects Likewise Exhibit New End Growth Polarity Errors

**Figure 6:**
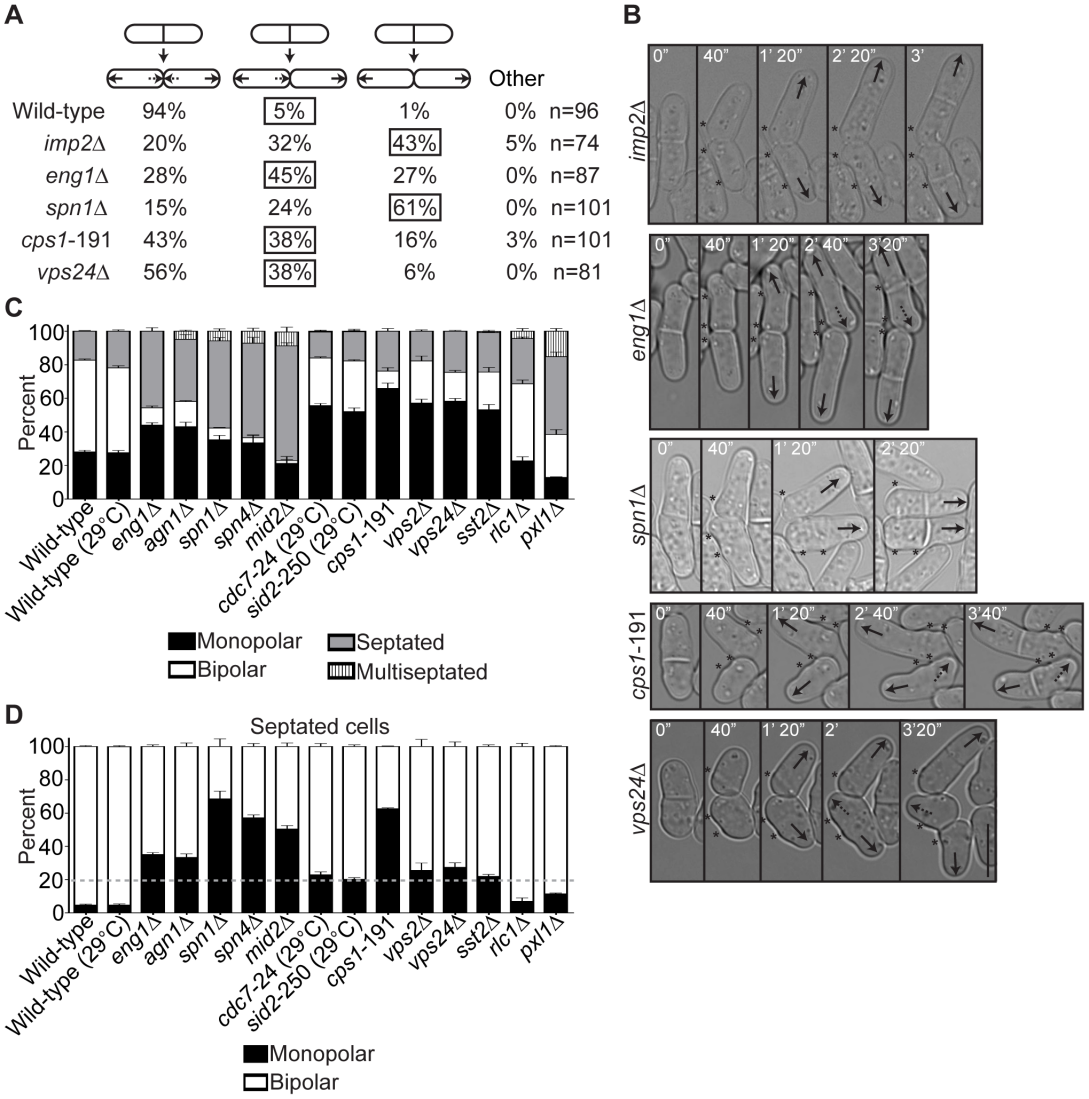
Late cytokinesis mutants phenocopy the new end-growth polarity defects of *fic1Δ* cells. (A) Quantification of growth patterns for cells of the indicated genotypes. Sample size (n) is provided for each genotype. The percentage of the most prevalent faulty growth pattern is boxed for each genotype. (B) Live-cell DIC movies of cells of the indicated genotypes scored in (A). The most prevalent faulty growth pattern for each genotype is pictured. Solid arrows denote old end growth, whereas dashed arrows indicate new end growth. Birth scars are marked by asterisks. Time points are noted. (C) Quantification of polarity phenotypes of calcofluor-stained cells of the indicated genotypes, with three trials per genotype and n>300 for each trial. Data are presented as mean ± SEM for each category. All cells were grown at 25°C unless otherwise noted. (D) Quantification of septated cells in (C), with three trials per genotype and n>200 for each trial. Data are presented as mean ± SEM for each category. A dashed gray line marks 20% on the y-axis (Bar = 5 µm).

Because faulty cytokinesis led to persistence of parts of the cell division machinery at *fic1*Δ division planes, we speculated that these remnants might deter subsequent polarized growth at new ends. If this were the case, one would expect other mutants with late cytokinesis defects to also show erroneous new end growth. Previous data had indicated that Fic1-associated Imp2 contributes to CR disassembly, with *imp2*Δ cells exhibiting abnormal actin structures flanking previous division sites [51]. Though we had shown that *imp2*Δ cells are defective in bipolar cell growth (Figure 4A–4C), we wanted to confirm that their growth defect was specific to new ends. Using time-lapse DIC imaging, we found that roughly 75% of *imp2*Δ cell divisions produced at least one daughter cell that failed at new end growth (Figure 6A). Interestingly, both *imp2*Δ daughter cells failed at new end growth in the majority of cases (Figure 6A–6B). Therefore, proper disassembly of CR components correlates with new end competency for polarized growth.

In addition to showing CR disassembly defects, *fic1*Δ cells also exhibited delays in septum remodeling at the division site. We therefore tested if disruption of septum degradation could likewise impact polarized growth. Loss of Eng1 or its cooperating glucanase, Agn1 [34], resulted in high percentages of monopolar growth (Figure 6C–6D and Figure S5A). Moreover, the growth defect of *eng1*Δ daughter cells was specific to new ends (Figure 6A–6B), and, similar to *fic1*Δ cells, *eng1*Δ daughter cells that initiated NETO prior to the next septation did so on average later than wild-type cells (129 min versus 75 min) (Figure S5B). Anillin-like Mid2 and the septin ring, of which Spn1 and Spn4 form the core [52], target these glucanases into a ring structure around septa [53]. Loss of any of these proteins likewise impaired bipolar cell growth (Figure 6C–6D and Figure S5A). In addition, though the majority of *spn1*Δ daughter cells failed at new end growth (Figure 6A–6B), those that initiated NETO prior to the next septation took longer on average to do so than wild-type cells (95 min versus 75 min) (Figure S5B). We therefore conclude that defective completion of cell wall remodeling at the division site, in addition to improper disassembly of CR components, compromises NETO efficiency.

The SIN coordinates many aspects of CR and septum regulation during late cytokinesis. Not only does SIN signaling oversee maintenance of a mature, homogenous CR [54], it mediates Cps1 targeting and accumulation at the division site [49], [50]. Loss of SIN signaling during cytokinesis can thus lead to CR fragmentation [54] and abortive septation [49]. Given these phenotypes and the synthetic genetic interactions between *fic1*Δ and SIN mutants (Figure S4A–S4B), we examined the relevance of the SIN to new end growth control. Temperature-sensitive alleles of genes encoding the SIN kinases Cdc7 and Sid2 caused mild but statistically significant growth polarity defects at semi-restrictive temperature (Figure 6C–6D and Figure S5A). A temperature-sensitive allele of the gene encoding Cps1, which functions downstream of the SIN, caused dramatic defects in establishing bipolar cell growth even at permissive temperature (Figure 6C–6D and Figure S5A). Additionally, a high proportion of *cps1*-191 cells failed specifically at new end growth (Figure 6A–6B), and those that were able to trigger NETO prior to subsequent septation did so on average later than wild-type cells (107 min versus 75 min) (Figure S5B). Not surprisingly, we were able to detect incomplete ingression of *cps1*-191 cells shifted to the restrictive temperature during cytokinesis (Figure S5C), again suggesting that these mutants experience remodeling errors at the division site.

Currently, the mechanism of membrane remodeling and scission at the *S. pombe* division site is unclear. In a variety of other organisms, endosomal sorting complex required for transport (ESCRT)-III factors contribute to this process [55]. ESCRT-III components have not been implicated in *S. pombe* cytokinesis regulation, though ESCRT-III-associated AMSH (*S. pombe* Sst2) localizes to the division site [56]. We found that deletions of genes encoding ESCRT-III components Vps2 and Vps24 or ESCRT-III-associated Sst2 were synthetically sick with a variety of loss-of-function cytokinesis alleles, including *imp2*Δ and *cps1*-191 (Figure S5D). Interestingly, loss of Vps2, Vps24, or Sst2 resulted in monopolar percentages significantly greater than observed for wild-type cells (Figure 6C–6D and Figure S5A), and nearly half of *vps24*Δ cell divisions resulted in one or both daughter cells that failed at new end growth prior to the next septation (Figure 6A–6B). Though these phenotypes were less penetrant than in other mutants, we speculate that ESCRT-III function guides membrane remodeling at the conclusion of *S. pombe* cell division to impact new end polarized growth.

Of note, deletion of *rlc1^+^* or paxillin *pxl1^+^*, which function primarily in early actomyosin function at the CR [47], [48], [57], [58], did not alter growth polarity percentages as significantly as other mutations or deletions (Figure 6C–6D and Figure S5A). Indeed, less than half of non-septated *rlc1*Δ and *pxl1*Δ cells were monopolar (Figure 6C), and the monopolar septated percentages of these genotypes were more similar to wild-type percentages than were those of the other mutants examined (Figure 6D). We therefore conclude that early steps in cytokinesis do not impact subsequent polarized cell growth as much as the terminal steps in cell division.

### Ineffective Cytokinesis Partially Impedes New End-Tip Growth Even upon Constitutive NETO Signaling

**Figure 7:**
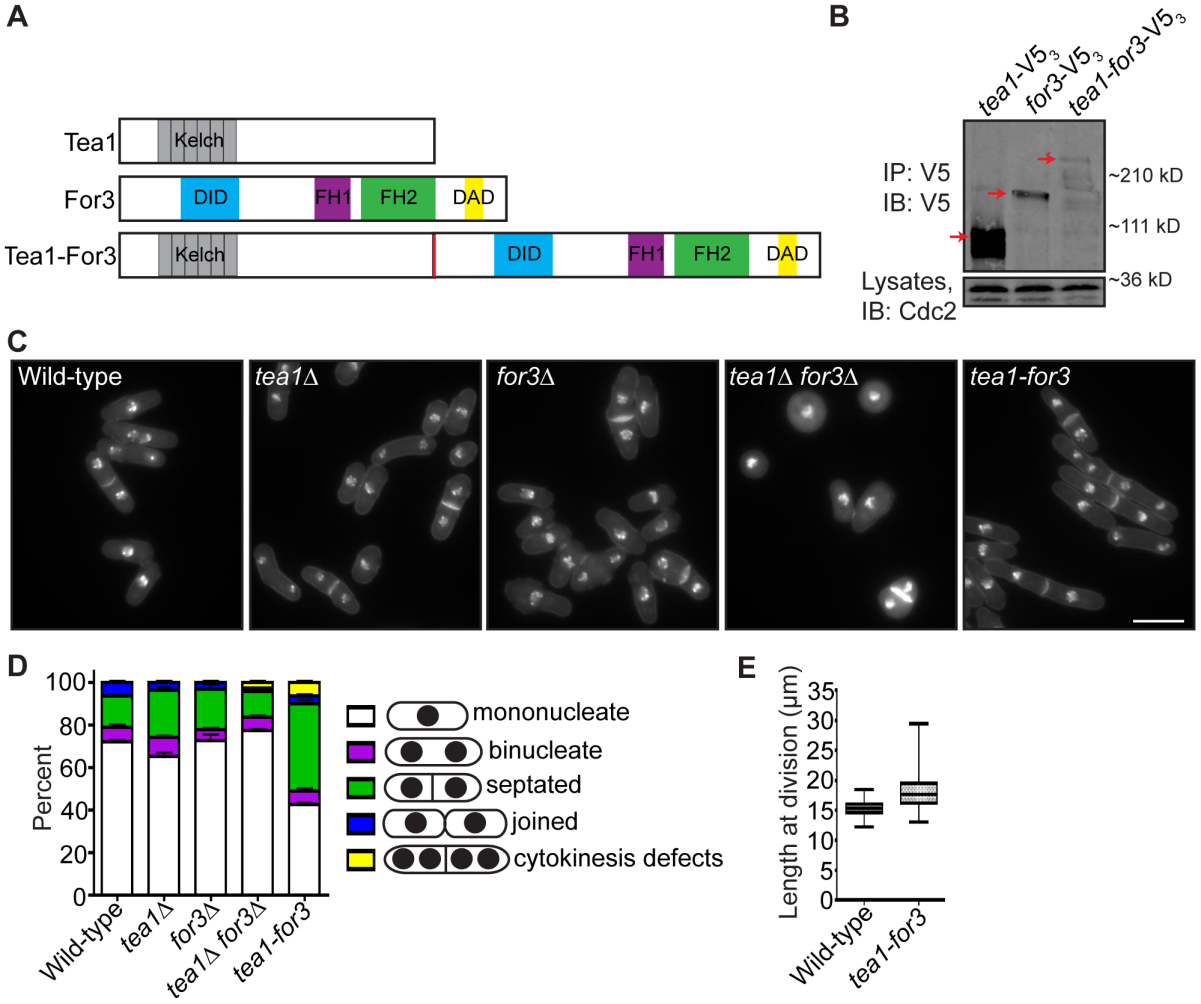
An endogenous Tea1-For3 fusion protein is functional but impinges on the cell division machinery. (A) Schematic of Tea1, For3, and Tea1-For3 protein domains and organization. (B) Anti-V5 immunoprecipitates from asynchronous *tea1*-V5_3_, *for3*-V5_3_, and *tea1-for3*-V5_3_ cells were blotted with anti-V5 antibodies. Arrows indicate full-length proteins. Lysates were blotted with anti-Cdc2 as a loading control. (C) Fixed-cell DAPI/methyl blue images of stained wild-type, *tea1Δ*, *for3Δ*, *tea1Δ for3Δ*, and *tea1-for3* cells. (D) Quantification of phenotypes of cells in (C), with three trials per genotype and n>300 for each trial. Data are presented as mean ± SEM for each category. (E) Quantification of cell lengths at cell division, with n>200 for each genotype. Data are presented as box-and-whisker plots showing the median (line in the box), 25^th^–75^th^ percentiles (box), and 5^th^–95^th^ percentiles (whiskers) for each genotype (Bar = 5 µm).

If faithful remodeling of the division site is important for growth competency of new ends, then one would expect that prematurely triggering NETO signaling just after cell division should not fully rescue the growth polarity defects of late cytokinesis mutants. To test this, we constructed a mutant that would undergo constitutive NETO. As over-expression of a fusion protein linking cell tip-associated Tea1 with formin For3 induces NETO in G1 [8], we integrated a Tea1-For3 fusion (Figure 7A) into the endogenous *tea1^+^* locus and deleted the single copy of the *for3^+^* gene. We confirmed that the Tea1-For3 fusion protein was produced *in vivo* (Figure 7B) and verified that this fusion was sufficient to induce NETO in a *cdc10*-V50 G1 arrest (Figure S6A–S6B). As previously reported [7], double deletion of *tea1^+^* and *for3* ^+^ resulted in general cell rounding (Figure 7C). However, expression of the Tea1-For3 fusion protein in the absence of Tea1 and For3 individually caused cells to regain their rod-shaped appearance (Figure 7C). Intriguingly, a high percentage of *tea1-for3* cells were either septated or exhibited cytokinesis defects (Figure 7C–7D), and *tea1-for3* cells were significantly longer at division than wild-type cells (on average, 18.3 µm versus 15.3 µm) (Figure 7C and 7E). Thus, though the endogenous Tea1-For3 fusion protein functioned in prematurely triggering NETO, it also affected cell division.

**Figure 8:**
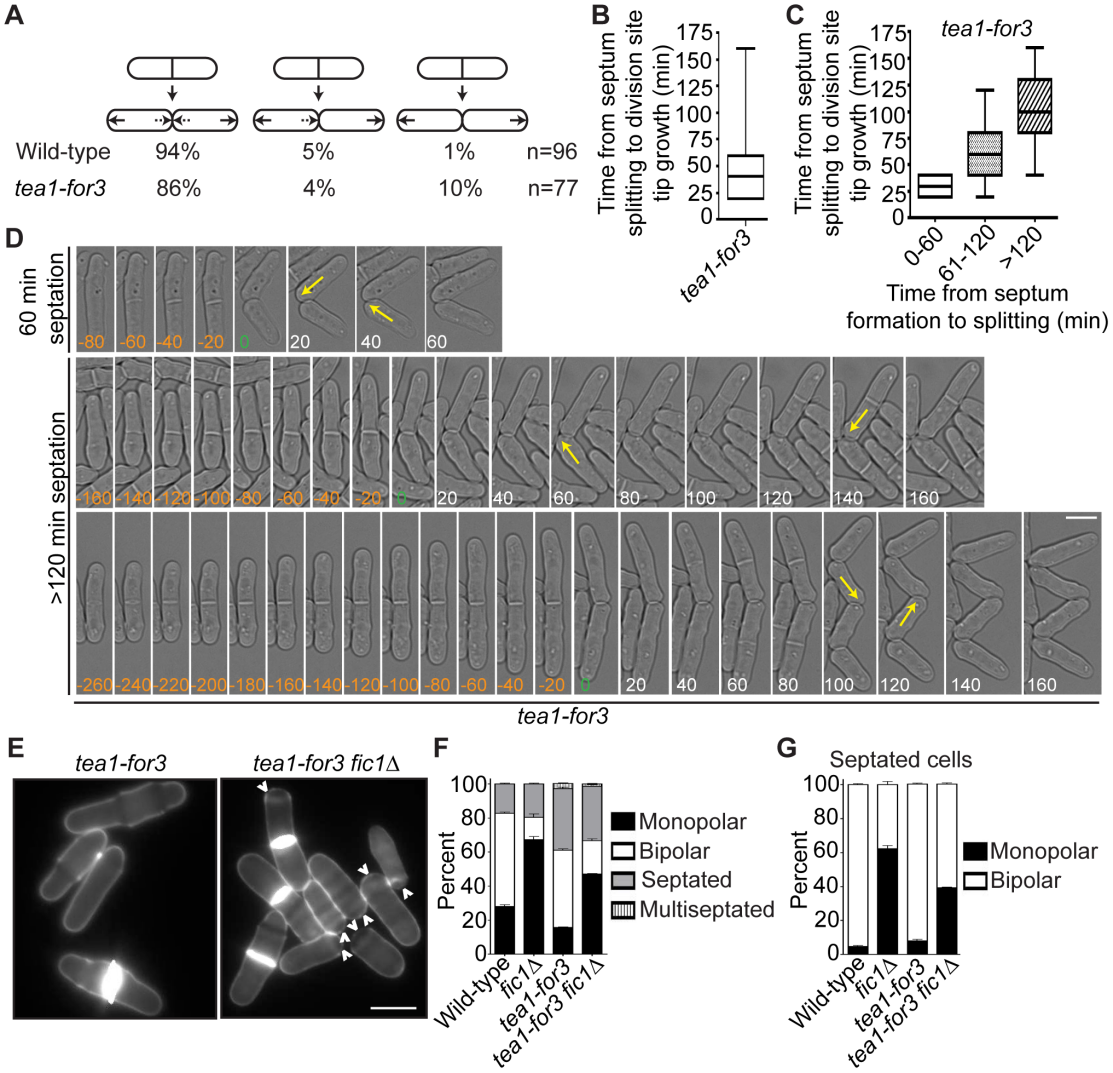
Constitutive NETO signaling does not fully rescue cytokinesis-based growth polarity defects. (A) Quantification of growth patterns for wild-type and *tea1-for3* cells. Sample size (n) is provided for each genotype. (B) Quantification of times from septum splitting to initiation of tip growth at previous division sites for *tea1-for3* cells. Times were carried into the next cell cycle where applicable. Data are presented in box-and-whisker plots showing the median (line in the box), 25^th^–75^th^ percentiles (box), and 5^th^–95^th^ percentiles (whiskers). n>200. (C) Data for *tea1-for3* cells in (B) grouped according to the amount of time needed for the mother cell to complete septation. Data are presented in box-and-whisker plots showing the median (line in the box), 25^th^–75^th^ percentiles (box), and 5^th^–95^th^ percentiles (whiskers) for each category. (D) Live-cell DIC movies of *tea1-for3* cells with different times needed to complete septation. The time of septum splitting of the mother cell is marked as point zero. The initiation of tip growth at previous division sites is denoted by yellow arrows. Tip growth at these sites was also scored for cells that did not initiate such growth until the subsequent cell cycle. (E) Live-cell images of calcofluor-stained *tea1-for3* and *tea1-for3 fic1Δ* cells. Arrowheads indicate monopolar cells. (F) Quantification of (E), with three trials per genotype and n>300 for each trial. Data are presented as mean ± SEM for each category. (G) Quantification of septated cells in (E) and (F), with three trials per genotype and n>200 for each trial. Data are presented as mean ± SEM for each category (Bars = 5 µm).

To analyze *tea1-for3* cells in real-time, we performed time-lapse DIC imaging. As expected, most *tea1-for3* cells underwent NETO before the next cell division (Figure 8A), with nearly 75% of new ends initiating growth within 50 minutes of septum splitting (Figure 8B). Nonetheless, some *tea1-for3* outliers took much longer to extend at tips created by cell division (Figure 8B). After grouping the times needed for tip growth to occur at previous division sites relative to the amount of time needed for the mother cell to complete cytokinesis, we found that newly-formed tips that took longer to initiate growth had been formed by more inefficient cytokinesis (Figure 8C–8D). As distal tip growth continued in cells undergoing division (Figure 8D) and appeared unimpeded by additional factors, these findings suggested that faulty cytokinesis imposes constraints at previous division sites that counteract positive polarizing cues. We corroborated this model by expressing the Tea1-For3 fusion in *fic1*Δ cells. Although *tea1-for3* cells were mostly bipolar, *tea1-for3 fic1*Δ cells showed a high percentage of monopolar growth (Figure 8E–8G). These findings confirmed that efficient completion of cytokinesis is critical for new end growth, even when signaling networks responsible for NETO are prematurely activated.

### Errors in Growth Polarity Caused by Faulty Cytokinesis Translate into Heightened Fungal Invasiveness

**Figure 9:**
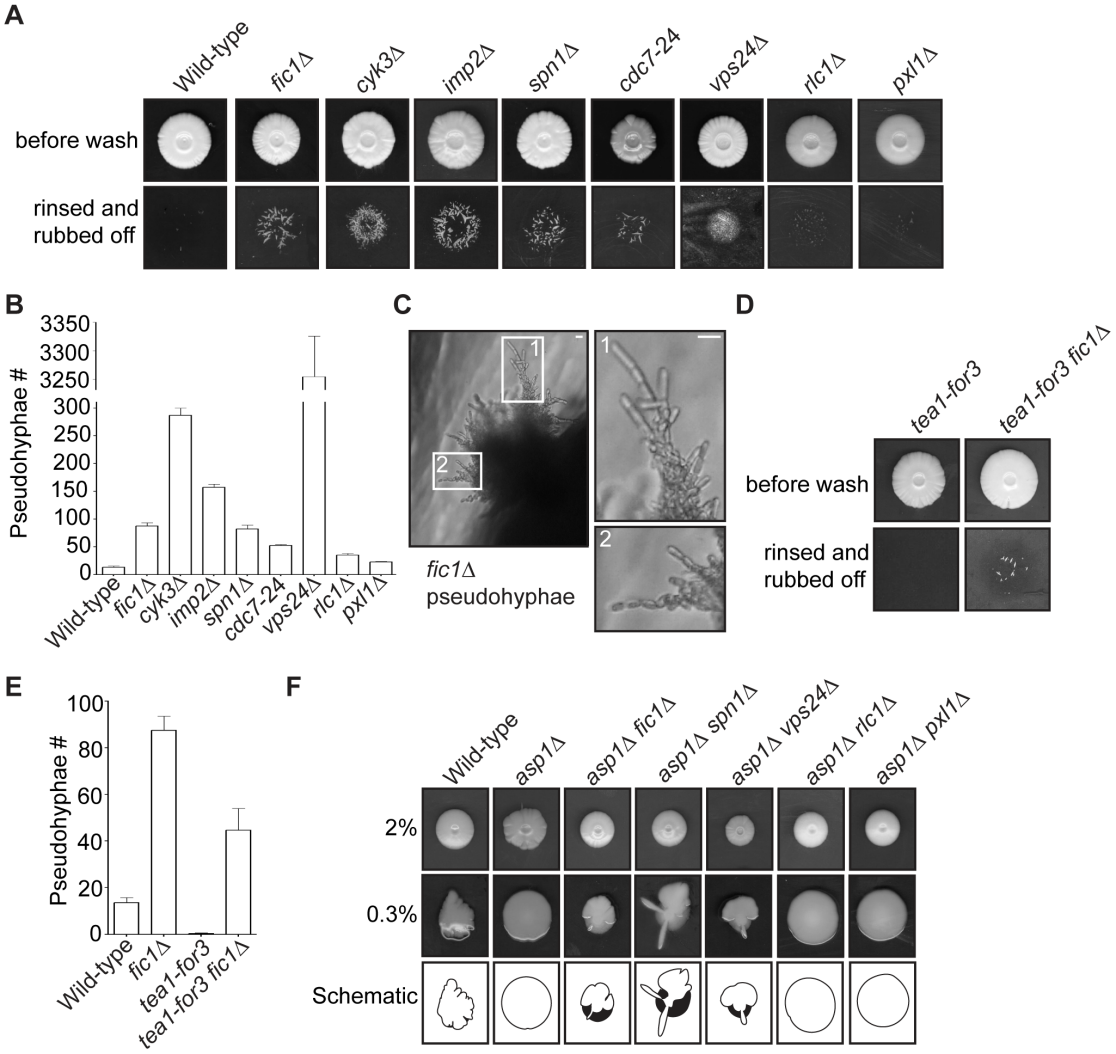
Cytokinesis mutants with growth polarity defects exhibit enhanced invasiveness. (A) Invasive growth assays for strains of the indicated genotypes on 2% agar. Cells were spotted on rich medium and incubated for 20 days at 29°C (top panel). Colonies were then rinsed under a stream of water and rubbed off (bottom panel). (B) Quantification of pseudohyphae in (A), with n≥3 for each genotype. Data are presented as mean ± SEM for each genotype. (C) Image of *fic1*Δ pseudohyphae in 2% agar, with enlarged images on the right. (D) Invasive growth assays for *tea1-for3* and *tea1-for3 fic1*Δ strains on 2% agar. Cells were spotted on rich medium and incubated for 20 days at 29°C (top panel). Colonies were then rinsed under a stream of water and rubbed off (bottom panel). (E) Quantification of pseudohyphae in (D), with n≥3 for each genotype. Data are presented as mean ± SEM for each genotype. (F) Colony growth of strains of the indicated genotypes on rich medium containing 2% (top panel) or 0.3% agar (middle panel). Cells were spotted and incubated for 12 days at 29°C. Schematics of colony growth on 0.3% agar are also given (bottom panel), with white areas representing growth on the agar surface and black areas representing growth into the agar (Bars = 5 µm).

*S. pombe* undergoing a dimorphic switch from single-celled to invasive form grow primarily in a monopolar fashion at old ends [39], [40]. Moreover, it has been postulated that cytokinesis errors might contribute to a hyphal-like transition in *S. pombe* [41]. We therefore considered that cytokinesis-based constraints on *S. pombe* growth polarity might facilitate invasive growth transitions. Using techniques similar to those described previously [40], [59], we tested whether various cytokinesis mutants displaying defective bipolar growth could form pseudohyphae into 2% agar. Cells lacking Fic1 or its interacting partners Cyk3 or Imp2 were significantly more invasive than wild-type cells (Figure 9A–9B). Like other invasive *S. pombe* mutants [39], [40], these mutants formed pseudohyphae composed of single cells oriented in filament-like projections (Figure 9C and Figure S7A). In addition to these strains, we found other cytokinesis mutants exhibiting high degrees of monopolar growth (*spn1*Δ, *cdc7*-24, and *vps24*Δ) to also be highly invasive and to form pseudohyphal projections into 2% agar (Figure 9A–9B and Figure S7A). Of note, the *vps24*Δ strain showed drastically more invasive growth than the others, though the reasons for this are currently unclear. *rlc1*Δ and *pxl1*Δ, which possess cytokinesis defects that do not considerably impact polarized cell growth (Figure 6C–6D and Figure S5A), invaded less efficiently on 2% agar than cytokinesis mutants exhibiting NETO defects (Figure 9A–9B). This supports the notion that defective cytokinesis promotes the dimorphic switch most robustly when it results in faulty NETO. As has previously been observed, *tea1*Δ also invaded well on 2% agar (Figure S7B–S7D). Thus, though cytokinesis-based constraints on growth polarity support enhanced *S. pombe* invasiveness, other polarity defects, which are not entirely specific to new ends, can do so as well.

Consistent with bipolar growth defects accompanying pseudohyphal growth, *tea1-for3* cells, which experience constitutive NETO induction, almost never extended pseudohyphae into 2% agar (Figure 9D–9E). Because cytokinesis-based constraints on growth polarity partially override tip-based NETO signaling, we reasoned that *tea1-for3* cells should become more invasive upon loss of Fic1. Indeed, on 2% agar *tea1-for3 fic1Δ* cells formed pseudohyphae (Figure S7E), which were more numerous than those observed for wild-type and *tea1-for3* strains (Figure 9D–9E). Thus, perturbations in cytokinesis cause growth polarity errors that facilitate pseudohyphal growth even upon constant NETO signaling.

Lastly, we asked whether loss of polarity-relevant cytokinesis factors could partially rescue invasiveness of an *asp1*Δ strain, which is unable to undergo the dimorphic switch due to an inability to sense external cues [40]. Previously, it was demonstrated that *asp1*Δ cells form a biofilm-like colony on 0.3% agar (Figure 9F) [40]. Growth on 0.3% agar is more sensitive for assaying invasiveness of strains that invade less efficiently, as wild-type colonies form protrusions on 0.3% agar but extend relatively few pseudohyphal projections into 2% agar (Figure 9A–9B and 9F) [40]. We therefore assessed the effect of cytokinesis defects on *asp1*Δ invasiveness by testing whether *asp1*Δ strains that also lacked relevant cytokinesis factors still formed biofilms on 0.3% agar. Intriguingly, double deletion strains of *asp1*Δ with *fic1*Δ, *spn1*Δ, or *vps24*Δ did not form biofilms on 0.3% agar but instead made projections into and on the surface of the agar (Figure 9F). In contrast, double deletion strains of *asp1D* with either *rlc1*Δ or *pxl1*Δ still formed biofilms on 0.3% agar (Figure 9F). We thus conclude that cytokinesis-based constraints on polarized cell growth in *S. pombe* can foster invasiveness even in the absence of typical nutritional signals.

## Discussion

**Figure 10:**
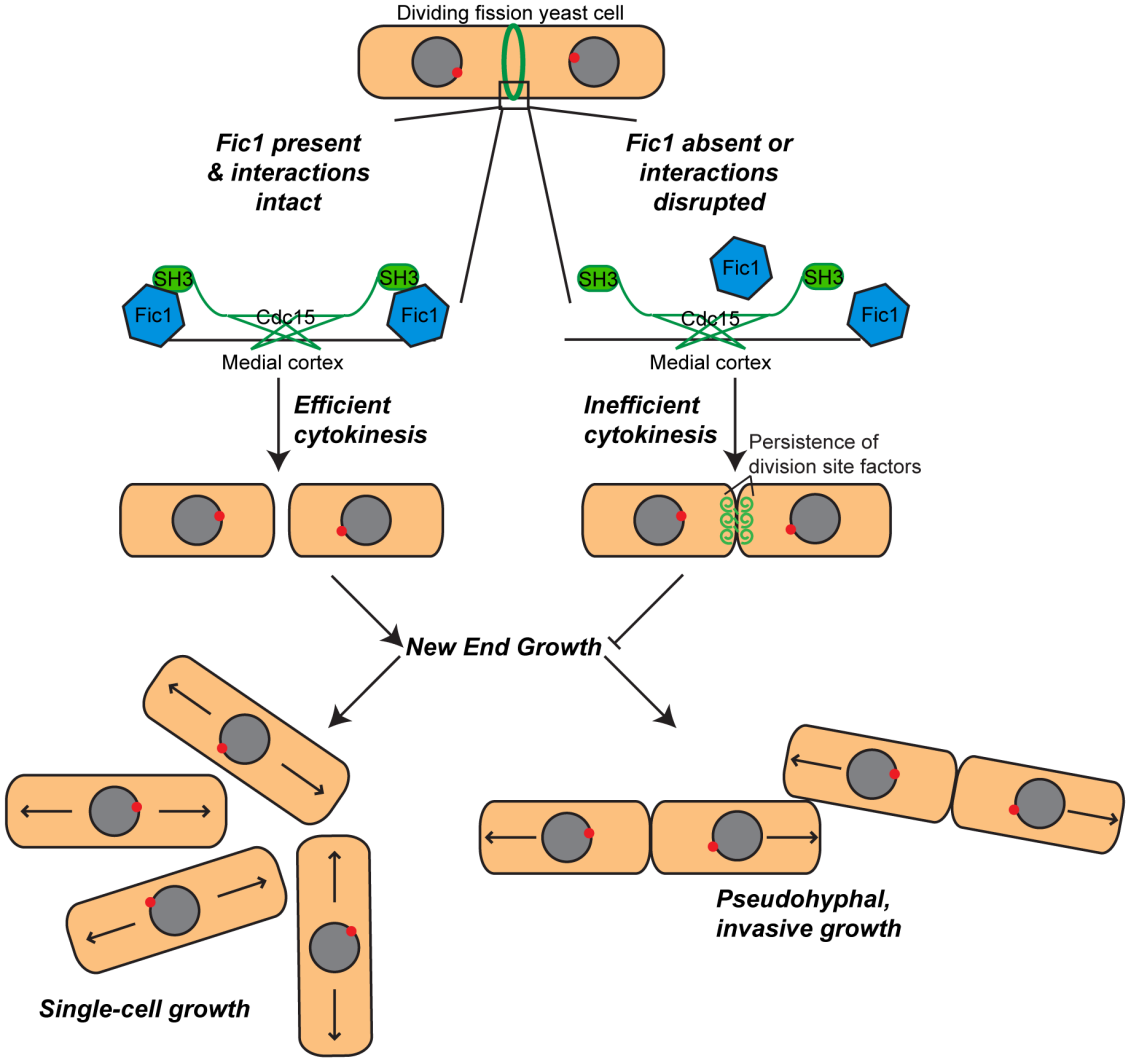
Model of Fic1's involvement in cytokinesis and the establishment of bipolar growth in *S.* **pombe**** **.** **** During cytokinesis, Fic1 serves as a scaffold for SH3 proteins, including Cdc15, at the cytokinetic ring. In the absence of Fic1, its interactions, or a parallel pathway, the completion of cell division is perturbed, and the cell division machinery persists at the previous division site. Failure to robustly complete cytokinesis impedes new end growth, even if NETO signaling is prematurely activated. Cytokinesis-based constraints on new end growth polarity aid in the transition into invasive fungal growth.

In this study, we have shown how cytokinesis and cell polarity crosstalk to regulate fission yeast morphogenesis. Our data support a model (Figure 10) in which Fic1 acts as an adaptor at the CR, where it guides proper completion of cytokinesis and thereby affects division site remodeling. Loss of Fic1, its interactions, or parallel pathways results in delayed growth at new ends, even upon constitutive activation of NETO signaling. Impaired bipolar cell growth resulting from defective cytokinesis in turn enhances *S. pombe* invasiveness.

### Cytokinesis-Based Regulation of Cell Polarity

The majority of *S. pombe* monopolar mutants previously analyzed fail at old end growth [4]. However, the cytokinesis mutants studied here were predominantly new end growth defective. As in other organisms [60], numerous *S. pombe* proteins known to affect growth polarity localize to the division site; this has fostered speculation that signaling at both cell tips and the division site might impact growth zones [23]. However, whether or not the cytokinesis functions of these proteins can specifically impact cell polarity has received little attention, especially in *S. pombe*. Our data provide evidence directly linking division site organization to *S. pombe* growth polarity. Because many factors involved in completing cell division likely also impact subsequent polarized growth, we believe our data could explain the involvement of diverse proteins in this process.

In other organisms, cytokinesis proteins appoint local regions of the cell cortex for growth following cell division [61]–[63]. For example, several budding yeast proteins, which remain at the cell cortex following cell division, have been reported to convey a cortical “tag” that marks the position of the next bud site [61], [62]. Similarly, during *Drosophila melanogaster* neurogenesis, cytokinetic furrow components mark the site from which the first dendrite will sprout [63]. In these cases, cytokinesis factors confer a positive polarizing cue adjacent to previous division sites, which contrasts with our findings in *S. pombe* where the cell division machinery impedes polarization and growth at new ends created by cell division.

The fact that *S. pombe* grow at old but not new ends after cell division is somewhat counterintuitive [4], especially because the cell growth machinery concentrates at the division site. Upon the completion of cytokinesis, the *S. pombe* growth machinery mysteriously shuttles to old ends rather than remaining at new ends. Why does the growth machinery relocate from the division site to old ends? One explanation is that new end cortices must be re-structured to become competent for tip growth. Indeed, specific lipid and cell wall variants contribute to *S. pombe* cytokinesis [64], [65], and local rearrangements of these may be required for growth activation. Moreover, the persistence of CR factors at new ends might create physical barriers to cytoskeletal elements, such as actin cables, required for tip growth. An inhibitory role for cell division in polarization is supported by studies of mutants that undergo multiple rounds of cytokinesis without physically separating, because internal cells in these structures do not grow into septa but branch adjacently. In a single-celled context, we speculate that when late cytokinetic events are perturbed, inherent delays in cortical re-structuring are exacerbated, causing growth polarity defects at new ends. In cases in which one daughter cell fails at NETO while the other is successful, we suspect these arise due to unequal partitioning of cytokinesis remnants and/or differences in cell cycle stages or life histories of daughter cells.

Consistent with furrow remodeling affecting polarization in other organisms, initial cellular protrusions of dividing mammalian cells orient away from the midbody linking daughter cells during abscission [66]. Only after the completion of cell division does polarization also occur near the latent division site [66]. Moreover, forced entry of HeLa cells with monopolar spindles into cytokinesis results in anucleate daughter cells that, similar to their nucleated counterparts, exhibit membrane protrusions only distal to cleavage furrows [67]. Thus, similar to our model in *S. pombe*, some factor at the division site cortex, and not a cell's cytosolic constituents, requires remodeling for post-cytokinetic polarization. Recent evidence indicates that mechanosensory pathways can direct cell polarization away from points of tension [68]. As modeling predicts that cortical tension peaks at the division plane during cytokinesis [69], it will likewise be important to assess the relevance of mechanical cues to cytokinesis-based polarization events.

### Interplay within NETO and Relevance of New End-Growth Control

Previous work has implied that association of microtubule-associated protein Tea1 with formin For3 at new ends is sufficient for NETO [8]. In our study, we expressed an endogenous Tea1-For3 fusion that could induce premature NETO. However, when cytokinesis was perturbed in *tea1-for3* mutants, NETO was delayed. We posit that local cortical abnormalities in cell wall, membrane, or associated factors can partially override typical growth cues in *S. pombe*, as has been observed in some plants [70]. Upon defective cytokinesis, such abnormalities at the division site may physically inhibit cell growth at new ends. These defects can lead to the formation of T-shaped cells when old end growth is also blocked, as in *tea1*Δ *fic1*Δ mutants. Our data underscore robust completion of cytokinesis as a major determinant of *S. pombe* NETO.

Is it beneficial for a cell to halt new end growth until well after cytokinesis completion? As mentioned previously, human cells undergoing division initially move away from each other, creating a pulling force that could contribute to abscission [66]. Highly adherent mammalian cells can actually complete cytokinesis, with some defects, in the absence of cortical myosin from the cleavage furrow [71]. Constriction-independent cytokinesis was first observed in the amoeba *Dictyostelium discoideum* [72], which accomplishes this task by likewise polarizing and growing distally to the division site [73]. One could imagine that in cases where *S. pombe* cell separation is delayed, tip growth at old ends might contribute similar forces to aid in abscission. Premature new end growth signaling might unbalance these forces, leading to exacerbated cytokinesis delays as in some *tea1-for3* cells. Premature new end growth might also interfere with remodeling during cytokinesis and thereby result in cell division defects. These findings highlight interdependence between the cell polarization and division machineries in *S. pombe*.

### Fic1 Scaffold Function during Cytokinesis

Our data indicate that Fic1's C terminus, and not its C2 domain, represents its major cytokinetic functional domain, contrasting with data reported for *S. cerevisiae* Inn1 [29], [30]. Why is Fic1's C2 domain dispensable for Fic1's cytokinesis, and thus polarity, functions? Sequence alignment indicates that there is in fact very low sequence identity between Fic1 and Inn1 C2 domains [30]. If Fic1's C2 domain is unable to perform functions or mediate interactions that Inn1's C2 domain can, it seems reasonable that Fic1-interacting proteins may be able to compensate. Consistent with this idea, over-expression of *S. cerevisiae* Cyk3 suppresses cytokinesis defects of *inn1*Δ mutants, suggesting Cyk3 function overlaps with Inn1 [29]. These data support that Fic1's C terminus is an efficient signaling platform, which scaffolds SH3 domain proteins through its PxxP motifs to ensure coherent integration of late cytokinesis signals.

What is the specific function of the Fic1 scaffold during cytokinesis? Our data indicate that loss of Fic1 leads to faulty CR disassembly and prolonged persistence of factors at the division site. CR disassembly defects were also observed in *inn1Δ S. cerevisiae* mutants, leading to speculation that Inn1 might stabilize the constricting CR by physically linking it to the ingressing membrane [30]. Subsequent findings countered that Inn1's C2 domain cannot bind phospholipids, and it was postulated instead that Inn1 cooperates with Cyk3 to coordinate cell wall deposition [29]. As in *fic1*Δ cells, septation and CR disassembly defects commonly accompany one another [51], [74]. Because these processes are inextricably linked, it is currently difficult to tease apart which defect precedes the other in *fic1*Δ cells. Moreover, completion of cytokinesis also requires lipid rearrangements in both animal cells and *S. pombe* [64], [75], and membrane bridges were observed in *fic1*Δ cells. We thus envision that Fic1's C terminus links signaling pathways that guide completion of multiple tasks during late cytokinesis and thereby affect new end remodeling. Of note, we believe that defects in early cytokinesis do not significantly alter bipolar growth establishment, as later defects more directly impinge on division site remodeling and have less time to be remedied before the next cell division. Our data furthermore support the notion that CR constriction and disassembly occur independently [74], as CR constriction but not disassembly proceeded appropriately in *fic1*Δ cells.

### Defective Cytokinesis in Invasive Fungal Growth

As fungal hyphae consist of long chains of cells, the transition into hyphal growth requires strict inhibition of cytokinesis. In some yeasts, Cdc14 phosphatase activates the Ace2 transcriptional program [76], which triggers expression of cell separation enzymes [77]. Upon the hyphal transition in *Candida albicans*, this signaling cascade is disrupted [78], and other transcription factors suppress expression of Ace2 targets [79]. Therefore, cytokinetic inhibition in hyphae is believed to operate largely on a transcriptional level, and reactivation of the Ace2 transcriptional program is thought to be responsible for the evolution of single-celled yeast growth [41].

In this study, we showed that fission yeast cells that undergo defective, yet not wholly abortive, cytokinesis exhibit enhanced invasive capacity. We believe cytokinesis-based constraints on growth polarity assist the transition into pseudohyphal growth because they force *S. pombe* to orient outwards and grow predominantly from old ends, a pattern commonly observed in *S. pombe* pseudohyphal growth [39], [40]. However, as demonstrated by *tea1*Δ cells, other changes in polarity can also enhance *S. pombe* invasiveness. Though not specifically defective at new end growth, *tea1*Δ cells grow predominantly in the direction of the mother cell, and these alterations in polarity might likewise favor growth orientations that are more conducive than bipolar growth to the invasive process.

We believe our data suggest that manipulation of cytokinesis proteins, and not necessarily signaling cascades that feed into downstream transcriptional pathways, can directly modulate the dimorphic switch. We thus speculate that the cytokinetic machinery might represent a direct target of the pseudohyphal developmental program. Intriguingly, loss of cytokinesis proteins that affect NETO rescued invasiveness of an *asp1*Δ mutant, which lacks the ability to detect nutritional cues [40] deemed important for the *S. pombe* dimorphic switch [36]. Because various environmental cues also regulate hyphal morphogenesis in pathogenic fungi [80], it will be important to assess the relative significance of cytokinesis-based controls on polarized growth for invasiveness in these species.

## Materials and Methods

### Strains and General Yeast Methods

The *S. pombe* strains used in this study (Table S1) were grown in either yeast extract (YE) or Edinburgh minimal media with relevant supplements. *fic1^+^*, *fic1N*, *fic1C*, *fic1-P257A*, *crn1^+^*, *tea4^+^*, *rlc1^+^*, *tea1^+^*, *for3* ^+^, and *tea1-for3* were tagged endogenously at the 3′ end with GFP:kan^R^, FLAG_3_:kan^R^, mCherry_3_:kan^R^, RFP:hyg^R^, V5:kan^R^, or V5:hyg^R^ cassettes as previously described [81]. A lithium acetate method [82] was used in *S. pombe* tagging transformations, and integration of tags was verified using whole-cell PCR and/or fluorescence microscopy. Introduction of tagged loci into other strains was accomplished using standard *S. pombe* mating, sporulation, and tetrad dissection techniques. For blocking of *cdc25*-22 strains in G2, cells were grown at 25°C and then shifted to 36°C for 3 h. For blocking of *nda3*-KM311 strains in prometaphase, cells were grown at 32°C and then shifted to 18°C for 6.5 h. For blocking of *cdc10*-V50 strains in G1, cells were grown at 25°C and then shifted to 36°C for 4 h. For blocking of *cps1*-191 cells in a cytokinesis arrest, cells were grown at 25°C and then shifted to 36°C for 3 h.

Mutants and truncations of *fic1* were expressed from the endogenous *fic1^+^* locus. To make these strains, a pIRT2 vector was originally constructed in which *fic1^+^* gDNA with 5′ and 3′ flanks was inserted between BamHI and PstI sites of pIRT2. Mutations were then introduced via site-directed mutagenesis. The *fic1(aa1-126)* construct was made by inserting a stop codon after residue 126. The *fic1(aa127-272)* construct was created by inserting XhoI sites before both the start codon and residue 127, digesting with XhoI to release the internal fragment, re-ligating the plasmid, and adding a start codon after the remaining XhoI site. *fic1*Δ was then covered by these pIRT2-*fic1* constructs, and stable integrants resistant to 5-FOA were isolated and confirmed by whole-cell PCR and western blotting.

To make the *tea1-for3* fusion, a pIRT2 vector was originally constructed in which *tea1^+^* gDNA with 5′ and 3′ flanks was inserted between SacI and SphI sites of pIRT2. Site-directed mutagenesis was performed to replace the *tea1^+^* stop codon with a SmaI/SalI/PstI multiple cloning site. *for3^+^* gDNA was amplified with a small N-terminal linker sequence and inserted between SmaI and PstI in this multiple cloning site (linker residues are Pro-Gly-Ade-Gly-Ade-Gly-Ade accounting for restriction site and added residues). *tea1*Δ was then covered by this pIRT2-*tea1-for3* construct, and stable integrants resistant to 5-FOA were isolated and confirmed by whole-cell PCR and western blotting. An integrant was subsequently mated with *for3Δ*, such that we could isolate *tea1-for3* strains in which *tea1^+^* and *for3* ^+^ were lacking.

Expression of acyl-GFP [83] was controlled by the thiamine-repressible *nmt1* promoter of pREP3 [84], [85]. Expression of LifeAct-GFP [86] was controlled by the thiamine-repressible *nmt81* promoter of pREP81 [87]. Expression from these *nmt* promoters was kept off by addition of 5 µg/mL thiamine to the medium, and expression was induced by washing and culturing in medium lacking thiamine for at least 24 h.

Spot assays to analyze genetic interactions were performed as previously described [88], except that all were done on YE agar. Synthetic interactions were judged based on differences in growth between double mutants and relevant single mutants.

### Yeast Two-Hybrid

Yeast two-hybrid analysis was performed as previously described [89], except that the bait and prey plasmids were either empty or encoded Cdc15 SH3 (aa843–927) [28], Cyk3 SH3 (aa1–59), wild-type [28] or mutant Fic1(aa190–269) fragments, or full-length Fic1.

### Protein Methods

Cells were lysed by bead disruption in NP40 lysis buffer in either native or denaturing conditions as previously described [90], except with the addition of 0.5 mM diisopropyl fluorophosphate (Sigma-Aldrich). Proteins were immunoprecipitated by anti-FLAG (Sigma-Aldrich), anti-Cdc15 [28], or anti-V5 (Invitrogen) antibodies. Immunoblot analysis of cell lysates and immunoprecipitates was performed using anti-FLAG, anti-Cdc15, anti-V5, anti-GFP (Roche), or anti-Cdc2 (Sigma-Aldrich) antibodies as previously described [88].

### Microscopy

Live-cell bright field images as well as all still images of cells expressing proteins endogenously-tagged with GFP, RFP, or mCherry were acquired on a spinning disc confocal microscope (Ultraview LCI; PerkinElmer) equipped with a 100X NA 1.40 PlanApo oil immersion objective, a 488-nm argon ion laser (GFP), and a 594-nm helium neon laser (RFP, mCherry). Images were taken via a charge-coupled device camera (Orca-ER; Hamamatsu Phototonics) and processed using Metamorph 7.1 software (MDS Analytical Technologies; Molecular Devices). Bright field images were used in determining cell lengths at division. Time-lapse GFP images of *cyk3*-GFP *sid4*-GFP cells secured on agar pads, which were sealed by Valap (a Vaseline, lanolin, and paraffin mixture), were acquired every 3 min using this system. Z-sections were acquired for all fluorescence images and combined into maximum projections. Cells were grown to log phase at 25°C before such imaging.

Images of yeast cells and pseudohyphae on YE agar plates were acquired by focusing a camera (PowerShot SD750; Canon) through a microscope (Universal; Carl Zeiss) equipped with a 20X NA 0.32 objective.

All other microscopy was performed using a personal DeltaVision microscope system (Applied Precision). This system includes an Olympus IX71 microscope, 60X NA 1.42 PlanApo and 100X NA 1.40 UPlanSApo objectives, fixed- and live-cell filter wheels, a Photometrics CoolSnap HQ2 camera, and softWoRx imaging software. The microscopy performed using this system was as follows:

For calcofluor staining, cells were washed in PBS and then resuspended in PBS containing 5 µg/mL calcofluor. After incubation on ice for 30 min, cells were washed three times in PBS and images were acquired using the personal DeltaVision system. Using the proximity of birth scars to cell ends, growth/morphology was scored as one of the following: monopolar (i.e., growth on one end), bipolar (i.e., growth on both ends), monopolar and septated, bipolar and septated, or multiseptated. For cells just completing division, daughter cells were scored as monopolar as long as ingression of the mother cell had progressed to such a degree that birth scars could be easily identified at new ends. All cells stained with calcofluor were grown to log phase at 25°C, except for the following: (1) *cdc25*-22 mutants, which were grown overnight at 25°C and then shifted to 36°C for 3 h before staining; (2) *cdc10*-V50 mutants, which were grown overnight at 25°C and then shifted to 36°C for 4 h before staining; and (3) SIN temperature-sensitive mutants and a wild-type control, which were grown overnight at 29°C before staining.
For live-cell DIC movies, movies of *rlc1*-GFP *sid4*-GFP cells, movies of GFP-*cps1 rlc1*-mCherry_3_ cells, and movies of *eng1*-GFP cells, cells were secured within the ONIX microfluidics perfusion system (CellASIC). Cells were loaded into Y04C plates for 5 sec at 8 psi, and YE liquid media flowed into the chamber at 5 psi during imaging. For the DIC movies, single z-planes were acquired every 20 min. Growth patterns were scored according to the tip growth that had occurred prior to the next septation. Relative cell ages were scored where applicable based on the number of birth scars seen on each daughter cell. Times until NETO were only determined for cells that initiated NETO prior to the next septation, except for *tea1-for3* cells, for which tip growth at new ends was monitored until growth at these sites occurred even if this carried into the next cell cycle. During time-lapse DIC imaging, daughter cells were judged as monopolar after septum splitting when birth scars could be identified, and timing for NETO was started at this point. The point of new end growth was noted when evident elongation occurred relative to birth scars formed by cytokinesis. For the fluorescence movies, multiple z-planes were acquired at different intervals, and z-planes were subsequently combined into maximum projections following deconvolution. Cells were grown to log phase at 25°C before acquisition of these movies.
For images of nuclei and cell walls in fixed cells, cells were fixed in 70% ethanol for at least 30 min and stained with DAPI and methyl blue. For counting of cells joined at their division sites, cells were also sonicated at 3.5 W following fixation to break weak associations. Single z-planes were acquired. Non-temperature-sensitive cells were grown to log phase at either 25°C or 32°C before imaging, and *cdc25*-22 mutants were grown overnight at 25°C and then shifted to 36°C for 3 h before fixation.
For acyl-GFP images, cells were either fixed or imaged live. *cdc25*-22 *fic1*Δ cells were grown overnight at 25°C in the presence of thiamine, grown an additional 21 h in the absence of thiamine to induce acyl-GFP expression, shifted to 36°C for 3 h before 70% ethanol fixation, washed three times in PBS, and then sonicated at 3.5 W to break weak associations. GFP z-planes were acquired, deconvolved, and combined into maximum projections. For acyl-GFP images of *cps1*-191 cells at restrictive temperature, cells were grown overnight at 25°C in the presence of thiamine, grown an additional 21 h in the absence of thiamine to induce acyl-GFP expression, and shifted to 36°C for 3 h before imaging.
For LifeAct-GFP images, cells were grown overnight at 25°C in the presence of thiamine and then grown an additional 30 h to induce expression of LifeAct-GFP. Single z-planes were acquired and deconvolved.
For phalloidin staining (adapted from [42]), cells were fixed in formaldehyde (Polysciences Inc.) for 5 min, and fixation was stopped by addition of PBS. Cells were washed three times in PBS and incubated with 0.1% NP40 for 1 min to permeablize cells. Cells were pelleted and washed three more times in PBS. Then, Alexa-Fluor 488 phalloidin (Molecular Probes) was added. Samples were placed on a nutator for 1 h and subsequently imaged. Images were processed using deconvolution.

### Invasive Growth Assays

To assay pseudohyphal invasion into 2% agar, 5 µl containing a total of 10^5^ cells were spotted on 2% YE agar and incubated at 29°C for 20 days. Colonies were subsequently placed under a steady stream of water and surface growth was wiped off using a paper towel. These methods were established in previous studies [40], [59].

To assay whether specific mutants rescued invasiveness of an *asp1*Δ strain on 0.3% agar [40], 1 µl containing 10^6^ cells was spotted on 0.3% YE agar as well as onto 2% agar as a control. Plates were incubated at 29°C for 12 days, at which point colony growth and/or biofilm formation were visualized.

## Supporting Information

Figure S1 Polarity and cytoskeletal defects of *fic1*Δ cells. (A) Schematic of phenotypes scored by calcofluor staining. Black bands represent birth scars. (B) Live-cell bright field (BF) and GFP images of *tea1*-GFP, *tea4*-GFP, *fic1Δ tea1*-GFP, and *fic1Δ tea4*-GFP cells. (C) Quantification of (B), with three trials per genotype and n>200 for each trial. Data are presented as mean ± SEM for each category. (D) Live-cell BF, GFP (in green), RFP (in magenta), and GFP/RFP merged images of *rgf1*-GFP *crn1*-RFP and *fic1Δ rgf1*-GFP *crn1*-GFP cells. (E) Live cell calcofluor (in magenta), GFP (in green), and calcofluor/GFP merged images of a calcofluor-stained *fic1Δ rgf1*-GFP cell. (F) Serial 10-fold dilutions of cells of the indicated genotypes. Cells were spotted on YE agar and incubated at 25°C, 29°C, 32°C, or 36°C. In the upper panel, all cells were spotted on the same plate for each temperature, though some intervening rows were removed in the figure presentation (Bars = 5 µm).
(TIF)

Figure S2 Fragments of Fic1 used for structure-function analysis. Lysates from cells of the indicated genotypes were blotted with an anti-GFP antibody, as well as with anti-Cdc2 as a loading control.
(TIF)

Figure S3 Analysis of Fic1-interacting proteins. (A) Quantification of monopolar Crn1-GFP in *cdc15ΔSH3 crn1*-GFP and *imp2Δ crn1*-GFP cells that were non-dividing or had only one division site. Three trials were performed per genotype, with n>100 for each trial. Data are presented as mean ± SEM. (B) Live-cell GFP images of *cdc15ΔSH3 crn1*-GFP and *imp2Δ crn1*-GFP cells scored in (A). Cells with monopolar Crn1-GFP are outlined with magenta dotted lines. (C) Schematic of Fic1 protein domain organization, with residues of interest marked, PxxP motifs (*) numbered, the region responsible for Cdc15 binding [28] indicated, and the sequence spanning the terminal two PxxPs given. (D) Yeast two-hybrid identification of the Cdc15 binding site on Fic1. *S. cerevisiae* strain PJ69-4A was co-transformed with bait and prey plasmids, which were either empty or expressed mutants/regions of Fic1 or Cdc15. P253A and P257A mutations were used to distinguish between PxxPs 10 and 11 as the motif responsible for Cdc15 binding. Two-hybrid interaction was judged by growth of transformants carrying both plasmids on selective media lacking histidine and adenine (-His, -Ade). None of the prey plasmids transactivated. (E) Live-cell bright field (BF), GFP (colored green), mCherry (mCh) (colored magenta), and GFP/mCh merged images of a *fic1*-GFP *cdc15*-mCherry interphase cell tip. (F) Live-cell BF and GFP images of *fic1-P257A*-GFP cells. Arrowheads mark Fic1 in CRs. (G) Yeast two-hybrid identification of Fic1 binding to Cyk3's SH3 domain. *S. cerevisiae* strain PJ69-4A was co-transformed with bait and prey plasmids, which were either empty or expressed Fic1 or Cyk3's SH3 domain. Two-hybrid interaction was judged by growth of transformants carrying both plasmids on selective media lacking histidine and adenine (-His, -Ade). None of the prey plasmids transactivated. (H) Live-cell BF and GFP images of *fic1-P257A*-GFP *imp2*Δ *cyk3*Δ cells. Arrowheads mark CR localization (Bars = 5 µm, except for S3E where Bar = 2 µm).
(TIF)

Figure S4 Analysis of cytokinesis defects of *fic1*Δ cells. (A) Serial 10-fold dilutions of cells of the indicated genotypes. Cells were spotted on YE agar plates that were incubated at 25°C, 27°C, 29°C, or 32°C. Mutation of *cdc16* causes SIN hyperactivation, whereas mutants of *spg1*, *cdc7*, or *sid2* exhibit loss of SIN function. *fic1*Δ was previously shown to be synthetically lethal with *sid2*-250 [28]. All cells were spotted on the same plate for each temperature, though some intervening rows were removed in the figure presentation. (B) *fic1*Δ and *spg1*-106 were mated, and tetrads were pulled on YE agar at 25°C. Genotypes were assessed by replica plating to YE agar at 36°C and to minimal medium lacking uracil. Images of colonies from a tetratype are also given. (C) Fixed-cell GFP images of G2-arrested *cdc25*-22 *fic1*Δ cells expressing acyl-GFP. Enlarged images of cells' division planes are also given. (D) Live-cell DIC, GFP (colored green), mCherry (mCh) (colored magenta), and GFP/mCh merged images of a *fic1*Δ *rlc1*-mCherry_3_ cell expressing LifeAct-GFP. Images are single z-planes. The solid white arrow in the GFP image indicates the division plane (which entirely lacks GFP signal), and the dashed white arrow in the GFP image indicates an abnormal actin mass flanking the division plane. (E) Live-cell bright field (BF) and GFP movies of *eng1*-GFP and *fic1*Δ *eng1*-GFP cells, with images acquired every 3 min. Representative images are shown for different times. Yellow arrows denote Eng1-GFP at the division site. (F) Quantification of times from ingression to Eng1-GFP disappearance from the division plane in movies scored in (E), with n>15 for each genotype. Data are presented in box-and-whisker plots showing the median (line in the box), 25^th^–75^th^ percentiles (box), and 5^th^–95^th^ percentiles (whiskers) for each genotype (Bars = 5 µm, except for enlarged regions in S4C where Bar = 2 µm).
(TIF)

Figure S5 Polarity and cytokinesis defects of late cytokinesis mutants. (A) Live-cell images of calcofluor-stained cells of the indicated genotypes (scored in Figure 6C–6D). Arrowheads indicate monopolar cells. For cells just completing division, daughter cells were scored as monopolar as long as ingression of the mother cell had progressed to such a degree that birth scars could be easily identified at new ends. (B) Quantification of times from septum splitting to initiation of growth at new ends in cells of the indicated genotypes that undergo NETO prior to the next septation in Figure 6A–6B. Data are presented as mean ± SEM for each genotype. (C) Live-cell DIC and GFP images of *cps1*-191 cells expressing acyl-GFP. Cells were shifted to 36°C for 3 h before imaging. Arrows indicate membrane bridges linking daughter cells. (D) Table of negative genetic interactions between deletion of genes encoding ESCRT-related proteins (ESCRT-III components Vps2 and Vps24, and ESCRT-III-associated deubiquitinase Sst2) and deletion/loss-of-function alleles of genes encoding cytokinesis factors (Imp2, myosin Myo2, β-glucan synthase Cps1, SIN GTPase Spg1, SIN kinase Sid2, and formin Cdc12) (Bars = 5 µm).
(TIF)

Figure S6 Premature NETO in *tea1-for3* cells. (A) Live-cell images of calcofluor-stained *cdc10*-V50 and *cdc10*-V50 *tea1-for3* cells arrested in G1. Arrowheads indicate monopolar cells. (B) Quantification of (A), with three trials per genotype and n>300 for each trial. Data are presented as mean ± SEM for each category (Bar = 5 µm).
(TIF)

Figure S7 Pseudohyphae of mutants with growth polarity defects. (A) Images of pseudohyphae for strains of the indicated genotypes in 2% agar. (B) Invasive growth assay for *tea1*Δ on 2% agar. Cells were spotted on rich medium and incubated for 20 days at 29°C (top panel). Colonies were then rinsed under a stream of water and rubbed off (bottom panel). (C) Quantification of pseudohyphae in (B), with n≥3 for each genotype. Data are presented as mean ± SEM for each genotype. Data for wild-type and *fic1*Δ strains are included for comparison. (D) Image of *tea1*Δ pseudohyphae in 2% agar. (E) Image of *tea1-for3 fic1Δ* pseudohyphae in 2% agar (Bars = 5 µm).
(TIF)

Table S1 *S. pombe* strains used in this study.
(DOC)
