# Correction: Cytokinesis-Based Constraints on Polarized Cell Growth in Fission Yeast

**DOI:** 10.1371/annotation/e5f64e2d-7587-43d1-a0af-bc6d6244889c

**Published:** 2013-08-29

**Authors:** K. Adam Bohnert, Kathleen L. Gould

In the originally published version of this manuscript, 'tea1∆' was sometimes erroneously changed to 'tea1;∆’. In the PDF, this resulted in deletion of a small portion of the adjacent text. This issue has now been fixed and the corrected HTML and PDF versions of the manuscript are now shown.

