# Supplementary figures and images for: Cytokinesis-Based Constraints on Polarized Cell Growth in Fission Yeast
Source: PLoS Genet. 2012 Oct 18;8(10):e1003004. doi: 10.1371/journal.pgen.1003004 (PMC3475658; doi:10.1371/journal.pgen.1003004)

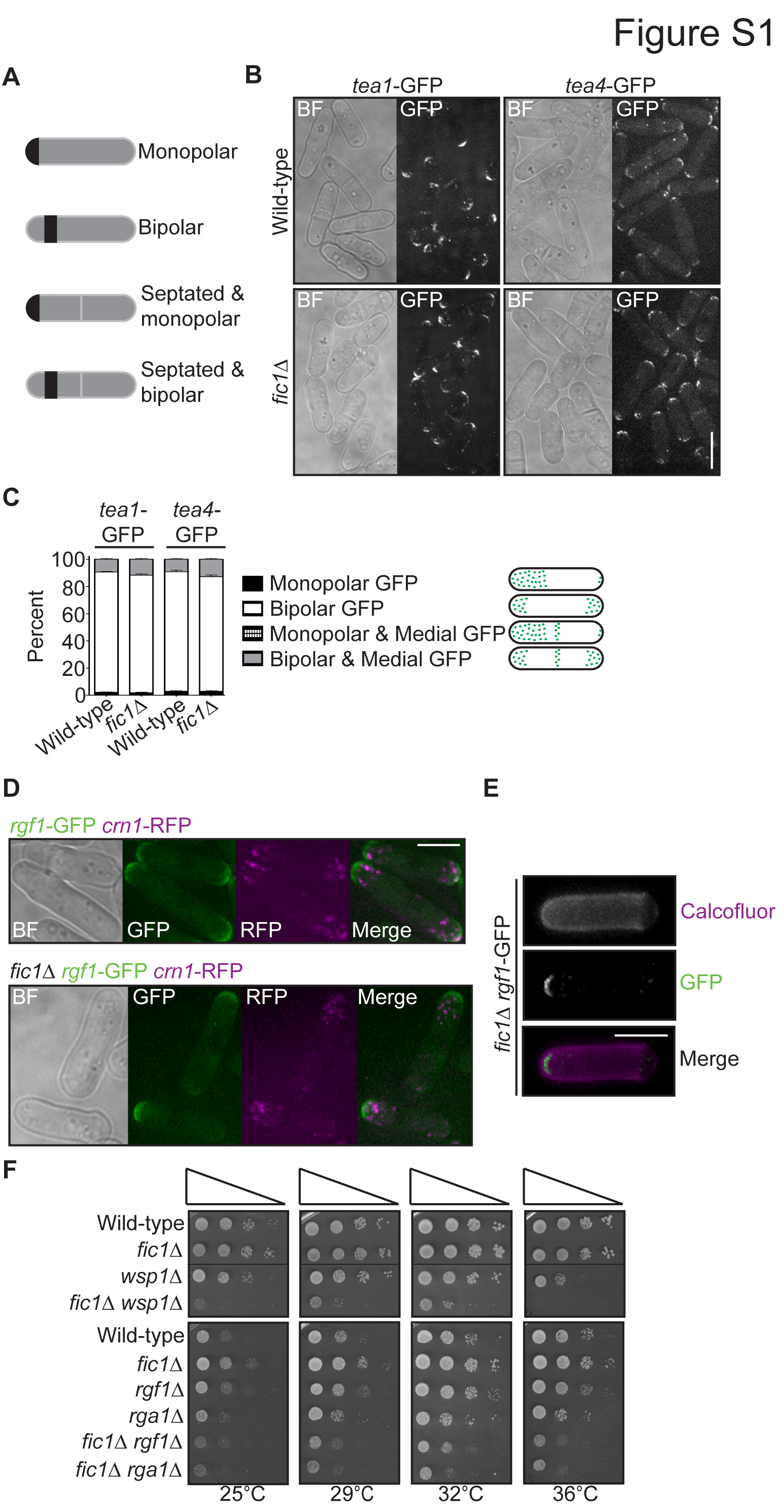

Supplement: Figure S1 — Polarity and cytoskeletal defects of fic1Δ cells. (A) Schematic of phenotypes scored by calcofluor staining. Black bands represent birth scars. (B) Live-cell bright field (BF) and GFP images of tea1-GFP, tea4-GFP, fic1Δ tea1-GFP, and fic1Δ tea4-GFP cells. (C) Quantification of (B), with three trials per genotype and n>200 for each trial. Data are presented as mean ± SEM for each category. (D) Live-cell BF, GFP (in green), RFP (in magenta), and GFP/RFP merged images of rgf1-GFP crn1-RFP and fic1Δ rgf1-GFP crn1-GFP cells. (E) Live cell calcofluor (in magenta), GFP (in green), and calcofluor/GFP merged images of a calcofluor-stained fic1Δ rgf1-GFP cell. (F) Serial 10-fold dilutions of cells of the indicated genotypes. Cells were spotted on YE agar and incubated at 25°C, 29°C, 32°C, or 36°C. In the upper panel, all cells were spotted on the same plate for each temperature, though some intervening rows were removed in the figure presentation (Bars = 5 µm). (TIF) [file pgen.1003004.s001.tif]

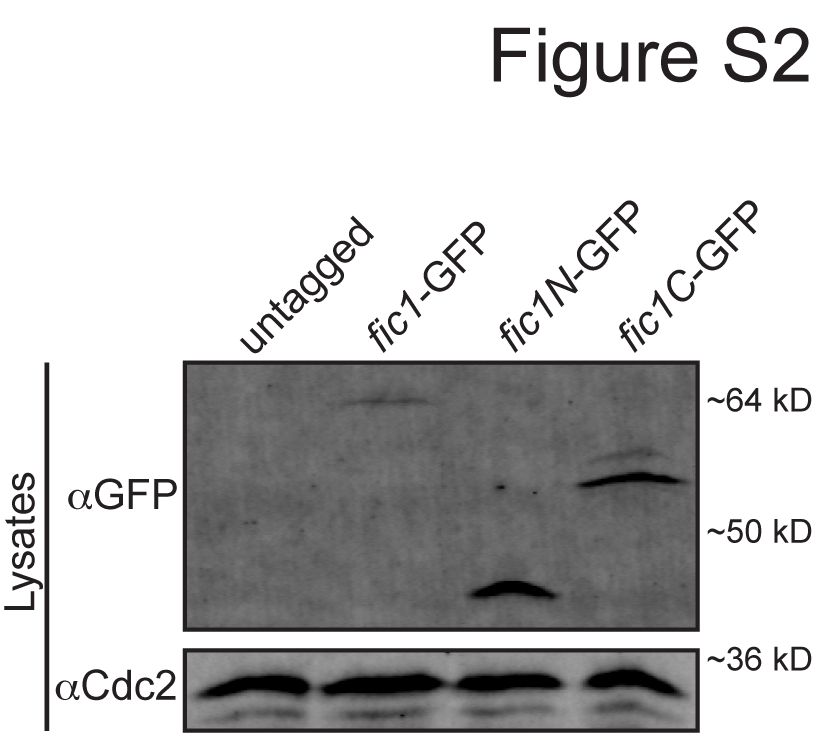

Supplement: Figure S2 — Fragments of Fic1 used for structure-function analysis. Lysates from cells of the indicated genotypes were blotted with an anti-GFP antibody, as well as with anti-Cdc2 as a loading control. (TIF) [file pgen.1003004.s002.tif]

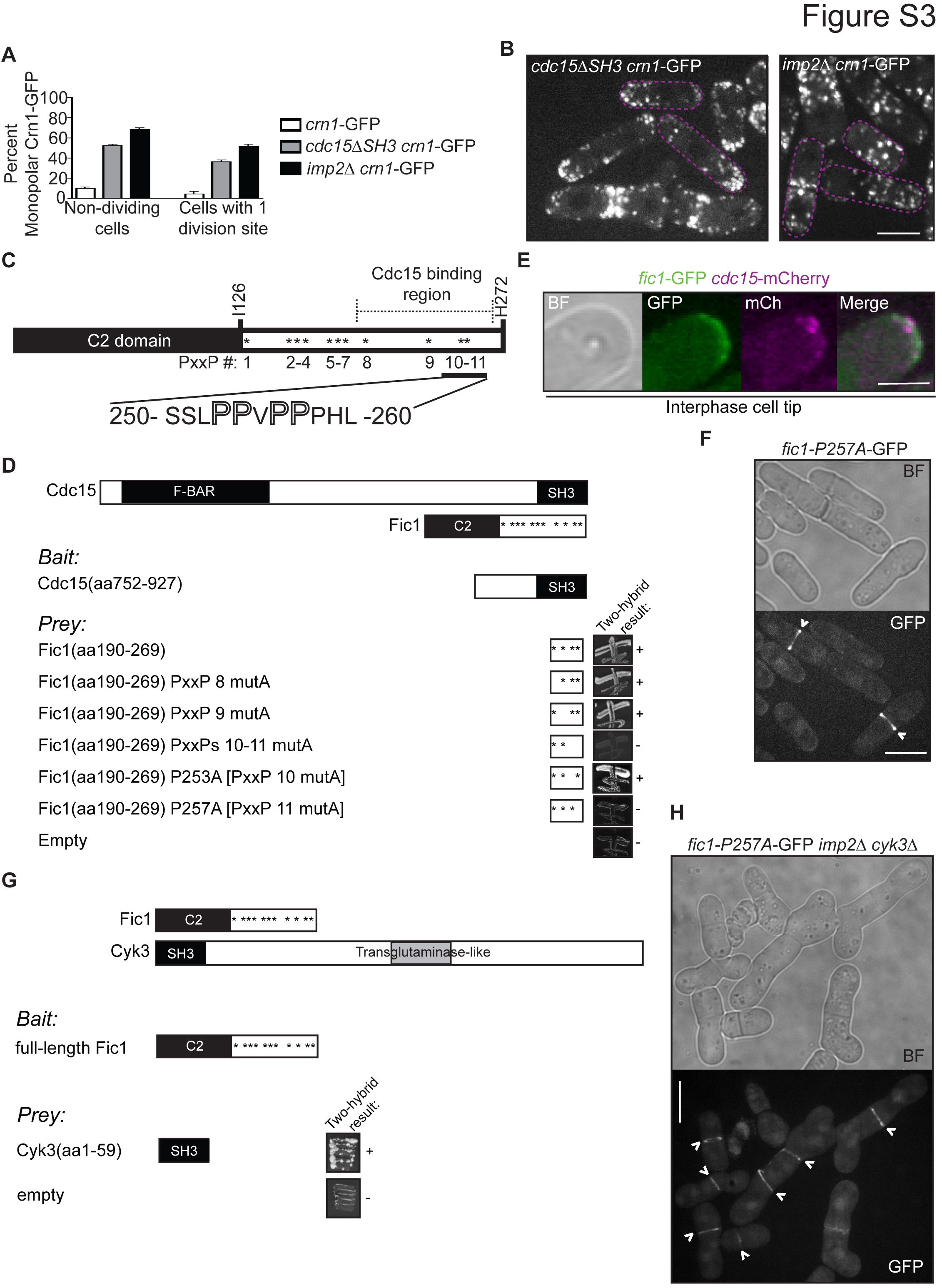

Supplement: Figure S3 — Analysis of Fic1-interacting proteins. (A) Quantification of monopolar Crn1-GFP in cdc15ΔSH3 crn1-GFP and imp2Δ crn1-GFP cells that were non-dividing or had only one division site. Three trials were performed per genotype, with n>100 for each trial. Data are presented as mean ± SEM. (B) Live-cell GFP images of cdc15ΔSH3 crn1-GFP and imp2Δ crn1-GFP cells scored in (A). Cells with monopolar Crn1-GFP are outlined with magenta dotted lines. (C) Schematic of Fic1 protein domain organization, with residues of interest marked, PxxP motifs (*) numbered, the region responsible for Cdc15 binding [28] indicated, and the sequence spanning the terminal two PxxPs given. (D) Yeast two-hybrid identification of the Cdc15 binding site on Fic1. S. cerevisiae strain PJ69-4A was co-transformed with bait and prey plasmids, which were either empty or expressed mutants/regions of Fic1 or Cdc15. P253A and P257A mutations were used to distinguish between PxxPs 10 and 11 as the motif responsible for Cdc15 binding. Two-hybrid interaction was judged by growth of transformants carrying both plasmids on selective media lacking histidine and adenine (-His, -Ade). None of the prey plasmids transactivated. (E) Live-cell bright field (BF), GFP (colored green), mCherry (mCh) (colored magenta), and GFP/mCh merged images of a fic1-GFP cdc15-mCherry interphase cell tip. (F) Live-cell BF and GFP images of fic1-P257A-GFP cells. Arrowheads mark Fic1 in CRs. (G) Yeast two-hybrid identification of Fic1 binding to Cyk3's SH3 domain. S. cerevisiae strain PJ69-4A was co-transformed with bait and prey plasmids, which were either empty or expressed Fic1 or Cyk3's SH3 domain. Two-hybrid interaction was judged by growth of transformants carrying both plasmids on selective media lacking histidine and adenine (-His, -Ade). None of the prey plasmids transactivated. (H) Live-cell BF and GFP images of fic1-P257A-GFP imp2Δ cyk3Δ cells. Arrowheads mark CR localization (Bars = 5 µm, except for S3E where Bar = 2 µm). (TIF) [file pgen.1003004.s003.tif]

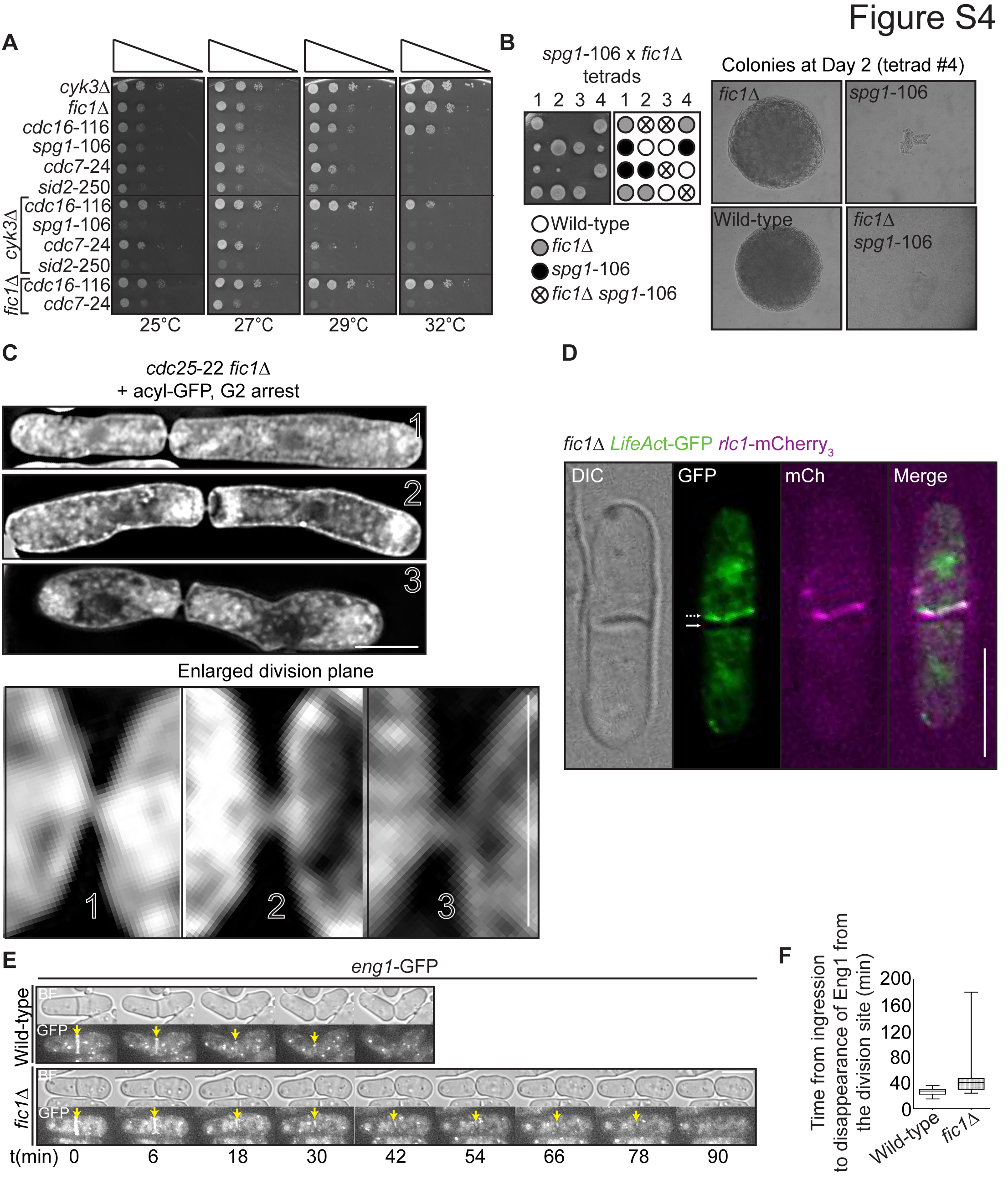

Supplement: Figure S4 — Analysis of cytokinesis defects of fic1Δ cells. (A) Serial 10-fold dilutions of cells of the indicated genotypes. Cells were spotted on YE agar plates that were incubated at 25°C, 27°C, 29°C, or 32°C. Mutation of cdc16 causes SIN hyperactivation, whereas mutants of spg1, cdc7, or sid2 exhibit loss of SIN function. fic1Δ was previously shown to be synthetically lethal with sid2-250 [28]. All cells were spotted on the same plate for each temperature, though some intervening rows were removed in the figure presentation. (B) fic1Δ and spg1-106 were mated, and tetrads were pulled on YE agar at 25°C. Genotypes were assessed by replica plating to YE agar at 36°C and to minimal medium lacking uracil. Images of colonies from a tetratype are also given. (C) Fixed-cell GFP images of G2-arrested cdc25-22 fic1Δ cells expressing acyl-GFP. Enlarged images of cells' division planes are also given. (D) Live-cell DIC, GFP (colored green), mCherry (mCh) (colored magenta), and GFP/mCh merged images of a fic1Δ rlc1-mCherry3 cell expressing LifeAct-GFP. Images are single z-planes. The solid white arrow in the GFP image indicates the division plane (which entirely lacks GFP signal), and the dashed white arrow in the GFP image indicates an abnormal actin mass flanking the division plane. (E) Live-cell bright field (BF) and GFP movies of eng1-GFP and fic1Δ eng1-GFP cells, with images acquired every 3 min. Representative images are shown for different times. Yellow arrows denote Eng1-GFP at the division site. (F) Quantification of times from ingression to Eng1-GFP disappearance from the division plane in movies scored in (E), with n>15 for each genotype. Data are presented in box-and-whisker plots showing the median (line in the box), 25th–75th percentiles (box), and 5th–95th percentiles (whiskers) for each genotype (Bars = 5 µm, except for enlarged regions in S4C where Bar = 2 µm). (TIF) [file pgen.1003004.s004.tif]

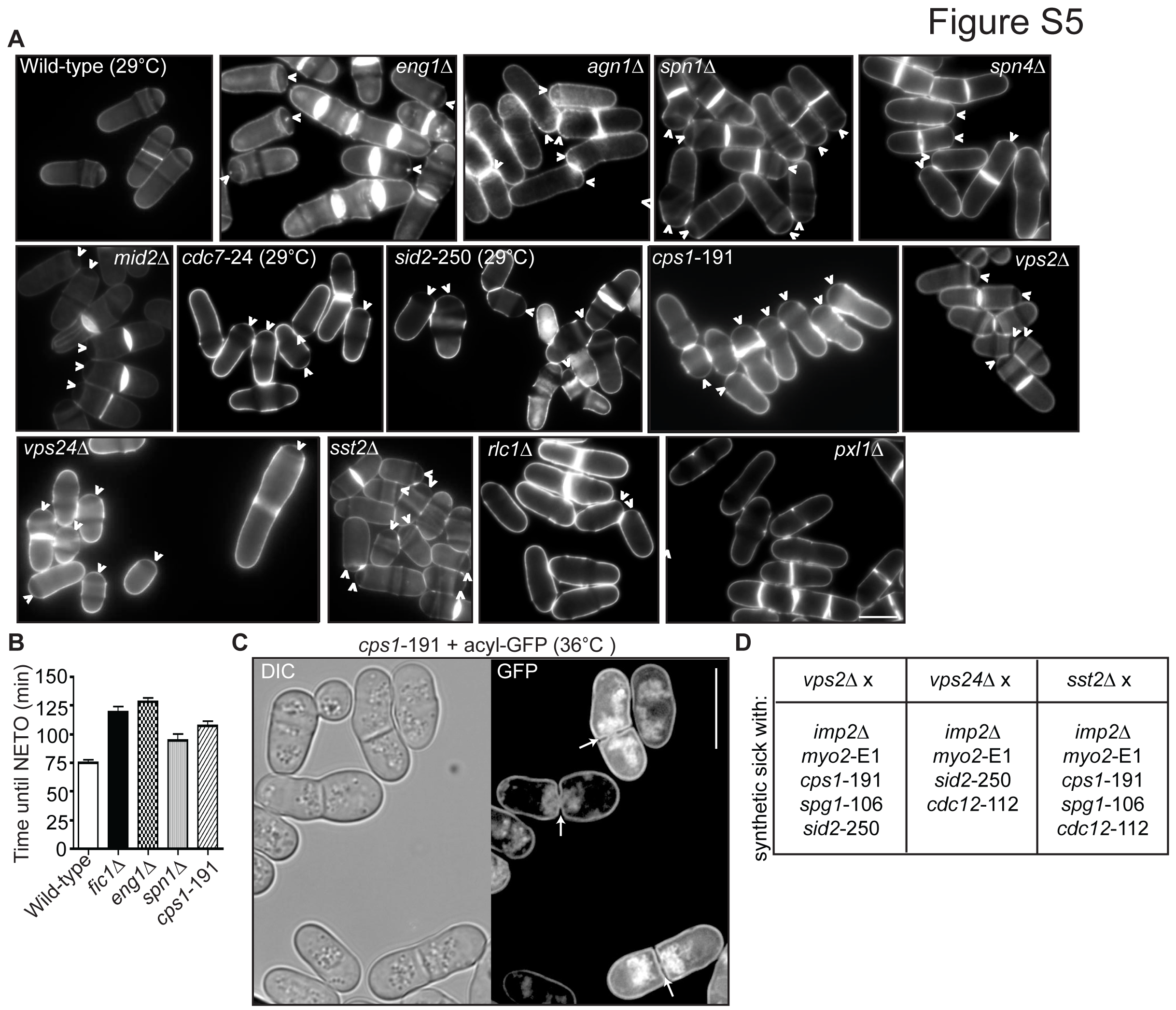

Supplement: Figure S5 — Polarity and cytokinesis defects of late cytokinesis mutants. (A) Live-cell images of calcofluor-stained cells of the indicated genotypes (scored in Figure 6C–6D). Arrowheads indicate monopolar cells. For cells just completing division, daughter cells were scored as monopolar as long as ingression of the mother cell had progressed to such a degree that birth scars could be easily identified at new ends. (B) Quantification of times from septum splitting to initiation of growth at new ends in cells of the indicated genotypes that undergo NETO prior to the next septation in Figure 6A–6B. Data are presented as mean ± SEM for each genotype. (C) Live-cell DIC and GFP images of cps1-191 cells expressing acyl-GFP. Cells were shifted to 36°C for 3 h before imaging. Arrows indicate membrane bridges linking daughter cells. (D) Table of negative genetic interactions between deletion of genes encoding ESCRT-related proteins (ESCRT-III components Vps2 and Vps24, and ESCRT-III-associated deubiquitinase Sst2) and deletion/loss-of-function alleles of genes encoding cytokinesis factors (Imp2, myosin Myo2, β-glucan synthase Cps1, SIN GTPase Spg1, SIN kinase Sid2, and formin Cdc12) (Bars = 5 µm). (TIF) [file pgen.1003004.s005.tif]

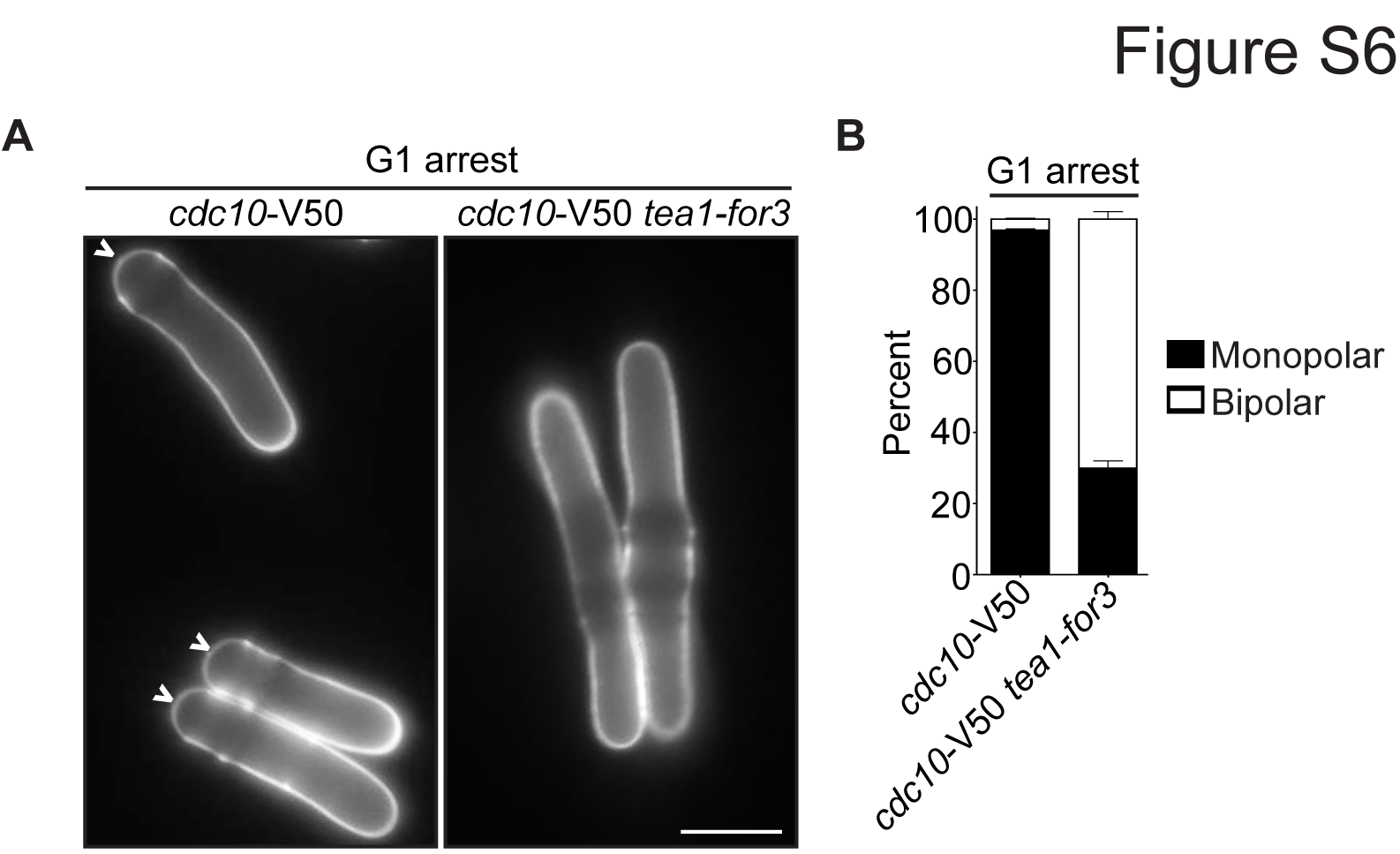

Supplement: Figure S6 — Premature NETO in tea1-for3 cells. (A) Live-cell images of calcofluor-stained cdc10-V50 and cdc10-V50 tea1-for3 cells arrested in G1. Arrowheads indicate monopolar cells. (B) Quantification of (A), with three trials per genotype and n>300 for each trial. Data are presented as mean ± SEM for each category (Bar = 5 µm). (TIF) [file pgen.1003004.s006.tif]

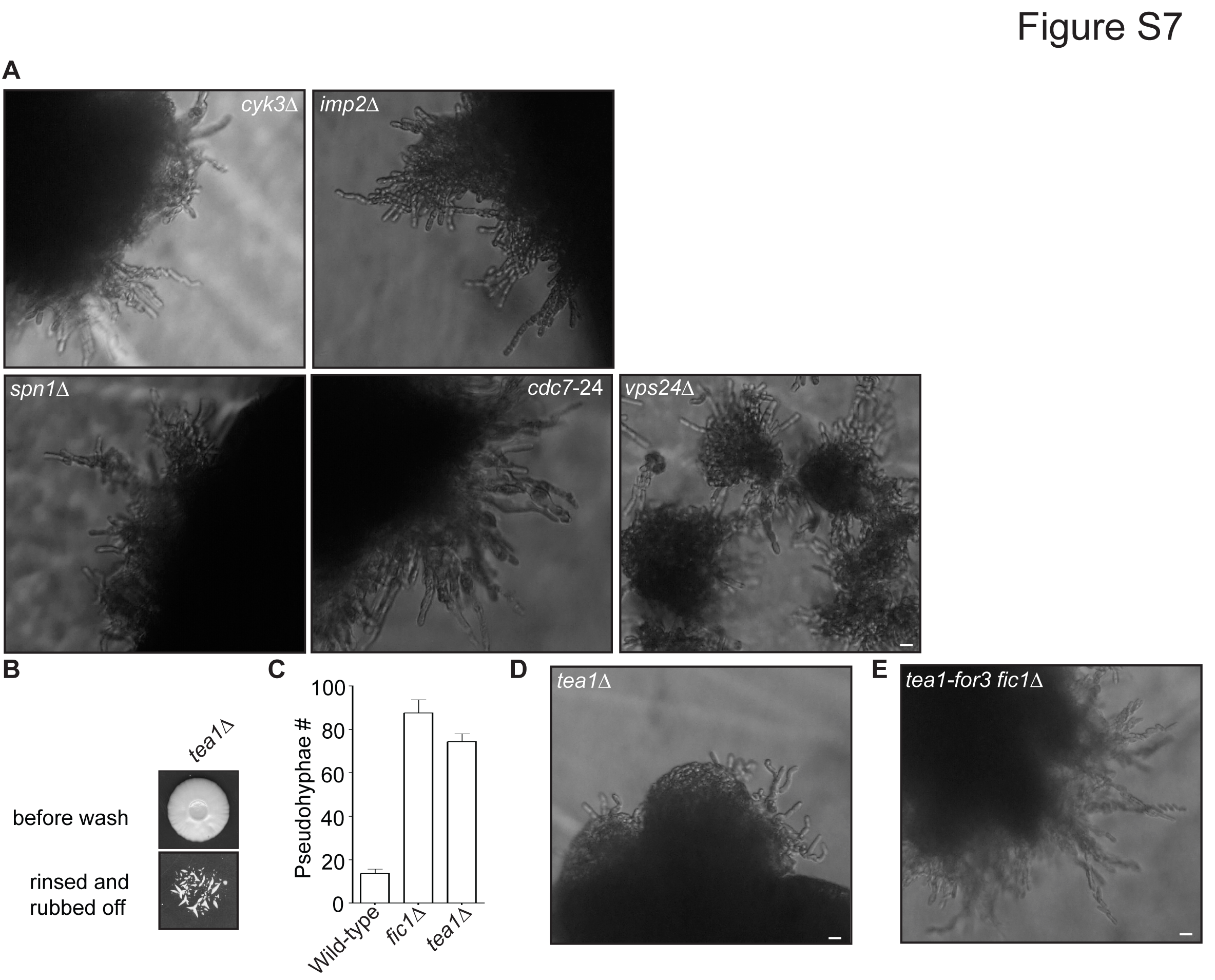

Supplement: Figure S7 — Pseudohyphae of mutants with growth polarity defects. (A) Images of pseudohyphae for strains of the indicated genotypes in 2% agar. (B) Invasive growth assay for tea1Δ on 2% agar. Cells were spotted on rich medium and incubated for 20 days at 29°C (top panel). Colonies were then rinsed under a stream of water and rubbed off (bottom panel). (C) Quantification of pseudohyphae in (B), with n≥3 for each genotype. Data are presented as mean ± SEM for each genotype. Data for wild-type and fic1Δ strains are included for comparison. (D) Image of tea1Δ pseudohyphae in 2% agar. (E) Image of tea1-for3 fic1Δ pseudohyphae in 2% agar (Bars = 5 µm). (TIF) [file pgen.1003004.s007.tif]
