# Supplementary material for: Cytokinesis-Based Constraints on Polarized Cell Growth in Fission Yeast
Source: PLoS Genet. 2012 Oct 18;8(10):e1003004. doi: 10.1371/journal.pgen.1003004 (PMC3475658; doi:10.1371/journal.pgen.1003004)
Supplement: Table S1 — S. pombe strains used in this study. (DOC) [file pgen.1003004.s008.doc]

**Table S1. *S. pombe* strains used in this study.**

| **#** | Genotype | Source |
| --- | --- | --- |
| **Figure 1** | | |
| KGY246 | *ade6-*M210 *leu1-*32 *ura4-*D18 h- | Lab stock |
| KGY6004 | *crn1*-GFP:kan^R^ *ade6-*M21X *leu1-*32 *ura4-*D18 h- | F. Chang |
| KGY6008 | *fic1::ura4^+^ ade6-*M21X *leu1-*32 *ura4-*D18 h+ | Lab stock |
| KGY10635 | *rgf1*-GFP:kan^R^ *sid4*-RFP:kan^R^ *ade6*-M21X *leu1*-32 *ura4*-D18 h- | This study |
| KGY10722 | *fic1::ura4^+^* *rgf1*-GFP:kan^R^ *sid4*-RFP:kan^R^ *ade6*-M21X *leu1*-32 *ura4*-D18 h+ | This study |
| KGY11148 | *fic1::ura4^+^ crn1*-GFP:kan^R^ *ade6*-M21X *leu1*-32 *ura4*-D18 h+ | This study |
| **Figure 2** | | |
| KGY246 | *ade6-*M210 *leu1-*32 *ura4-*D18 h- | Lab stock |
| KGY852 | *cdc25*-22 *ade6*-M216 *leu1*-32 *ura4*-D18 h+ | Lab stock |
| KGY6008 | *fic1::ura4^+^ ade6-*M21X *leu1-*32 *ura4-*D18 h+ | Lab stock |
| KGY9065 | *tea1::ura4^+^* *leu1*-32 *ura4*-D18 h- | P. Nurse |
| KGY10780 | *fic1::ura4^+^* *cdc25*-22 *ade6*-M21X *leu1*-32 *ura4*-D18 h- | This study |
| KGY11092 | *tea1::ura4^+^* *fic1::ura4^+^* *leu1*-32 *ura4*-D18 h- | This study |
| **Figure 3** | | |
| KGY11213 | *fic1N* *ade6*-M210 *leu1*-32 *ura4*-D18 h- | This study |
| KGY11308 | *fic1C* *ade6*-M210 *leu1*-32 *ura4*-D18 h+ | This study |
| KGY11313 | fic1C-GFP:kan^R^ *ade6*-M210 *leu1*-32 *ura4*-D18 h+ | This study |
| KGY11859 | *fic1C-*GFP:kan^R^ *cdc15*-mCherry:kan^R^ *sid4*-GFP:kan^R^ *ade6*-M21X *leu1*-32 *ura4*-D18 h+ | This study |
| KGY11860 | *fic1N-*GFP:kan^R^ *cdc15*-mCherry:kan^R^ *sid4*-GFP:kan^R^ *ade6*-M21X *leu1*-32 *ura4*-D18 h+ | This study |
| KGY12658 | *fic1*-GFP:kan^R^ *ade6*-M210 *leu1*-32 *ura4*-D18 h- | This study |
| KGY13233 | *fic1-*GFP:kan^R^ *cdc15*-mCherry:kan^R^ *sid4*-GFP:kan^R^ *ade6*-M21X *leu1*-32 *ura4*-D18 h+ | This study |
| **Figure 4** | | |
| KGY246 | *ade6*-M210 *leu1*-32 *ura4*-D18 h- | Lab stock |
| KGY3354 | *imp2::ura4^+^ ade6*-M216 *leu1*-32 *ura4*-D18 h+ | S. Sazer |
| KGY5637 | *nda3*-KM311 *ade6*-M210 *leu1*-32 *ura4*-D18 h- | Lab stock |
| KGY6373 | *fic1*-FLAG_3_:kan^R^ *ade6*-M21X *leu1*-32 *ura4*-D18 h+ | Lab stock |
| KGY6659 | *cdc25*-22 *fic1*-FLAG_3_:kan^R^ *ade6*-M21X *leu1*-32 *ura4*-D18 h- | Lab stock |
| KGY7051 | *cdc15∆SH3* *ade6*-M21X *leu1*-32 *ura4*-D18 h- | Lab stock |
| KGY7125 | *nda3*-KM311 fic1-FLAG_3_:kan^R^ *ade6*-M21X *leu1*-32 *ura4*-D18 h- | Lab stock |
| KGY7143 | *cyk3::ura4^+^* *ade6*-M21X *leu1*-32 *ura4*-D18 h+ | Lab stock |
| KGY7286 | *nda3*-KM311 *cyk3*-FLAG_3_:kan^R^ *ade6*-M21X *leu1*-32 *ura4*-D18 h+ | Lab stock |
| KGY7970 | *cyk3*-GFP:kan^R^ *sid4*-GFP:kan^R^ *ade6*-M21X *leu1*-32 *ura4*-D18 h- | Lab stock |
| KGY11857 | *fic1-P257A* *ade6*-M21X *leu1*-32 *ura4*-D18 h+ | This study |
| KGY11876 | *fic1-P257A*-FLAG_3_:kan^R^ *ade6*-M21X *leu1*-32 *ura4*-D18 h+ | This study |
| KGY11913 | *nda3*-KM311 *fic1-*V5_3_:kan^R^ *cyk3*-FLAG_3_:kan^R^ *ade6*-M21X *leu1*-32 *ura4*-D18 h- | This study |
| KGY12063 | *nda3*-KM311 *fic1*-V5_3_:kan^R^ *ade6*-M21X *leu1*-32 *ura4*-D18 h+ | This study |
| **Figure 5** | | |
| KGY246 | *ade6-*M210 *leu1-*32 *ura4-*D18 h- | Lab stock |
| KGY852 | *cdc25*-22 *ade6*-M216 *leu1*-32 *ura4*-D18 h+ | Lab stock |
| KGY6008 | *fic1::ura4^+^ ade6-*M21X *leu1-*32 *ura4-*D18 *h+* | Lab stock |
| KGY7451 | *rlc1*-GFP: *ura4^+^ sid4*-GFP:kan^R^ *ade6*-M21X *leu1*-32 *ura4*-D18 h+ | Lab stock |
| KGY10780 | *fic1::ura4^+^* *cdc25*-22 *ade6*-M21X *leu1*-32 *ura4*-D18 h- | This study |
| KGY12026 | *fic1::ura4^+^ rlc1*-GFP:*ura4^+^ sid4*-GFP:kan^R^ *ade6*-M21X *leu1*-32 *ura4*-D18 h- | This study |
| KGY13347 | *fic1::ura4^+^* GFP*-cps1*:kan^R^ *rlc1*-mCherry_3_:kan^R^ *leu1*-32 *ura4*-D18 h- | This study |
| KGY13348 | GFP*-cps1*:kan^R^ *rlc1*-mCherry_3_:kan^R^ *leu1*-32 *ura4*-D18 h- | This study |
| **Figure 6** | | |
| KGY246 | *ade6*-M210 *leu1*-32 *ura4*-D18 h- | Lab stock |
| KGY925 | *spn4::ura4^+^ ade6-*M21X *leu1*-32 *ura4­*-D18 h- | Lab stock |
| KGY1105 | *sid2*-250 *ade6*-M21X *leu1*-32 *ura4*-D18 h- | Lab stock |
| KGY1546 | *agn1::ura4^+^ade6-*M21X *leu1*-32 *ura4*-D18 h+ | Lab stock |
| KGY2153 | *cps1*-191 *ade6*-M210 *ura4*-D18 *lys1*-131 h- | Lab stock |
| KGY3135 | *mid2::ura4^+^ade6*-M21X *leu1*-32 *ura4*-D18 h- | Lab stock |
| KGY3354 | *imp2::ura4^+^ ade6*-M216 *leu1*-32 *ura4*-D18 h+ | S. Sazer |
| KGY4320 | *spn1::ura4^+^ ade6-*M21X *leu1*-32 *ura4*-D18 h+ | Lab stock |
| KGY5032 | *eng1::*kan^R^ *ura4*-D18 h- | Lab stock |
| KGY6628 | *cdc7*-24 *ade6*-M21X *leu1*-32 *ura4*-D18 h+ | Lab stock |
| KGY6658 | *rlc1::ura4^+^ ade6*-M21X *leu1*-32 *ura4*-D18 h- | Lab stock |
| KGY7870 | *sst2::ura4^+^ ade6*-M210 *leu1*-32 *ura4*-D18 h- | Lab stock |
| KGY8214 | *pxl1::*kan^R^ *ade6*-M21X *leu1*-32 *ura4*-D18 h+ | Lab stock |
| KGY12869 | *vps2::LEU2^+^* *leu1-*32 *ura4-*C190T h- | K. Takegawa |
| KGY12872 | *vps24::ura4^+^ ade6-*M21X *leu1*-32 *ura4-*D18 h- | K. Takegawa |
| **Figure 7** | | |
| KGY246 | *ade6*-M210 *leu1*-32 *ura4*-D18 h- | Lab stock |
| KGY5325 | *for3::*kan^R^ *ade6*-M21X *leu1*-32 *ura4*-D18 h- | F. Chang |
| KGY9065 | *tea1::ura4^+^ leu1*-32 *ura4*-D18 h- | P. Nurse |
| KGY13496 | *tea1::ura4^+^ for3::*kan^R^ *ade6*-M21X *leu1*-32 *ura4*-D18 h- | This study |
| KGY13592 | *tea1::tea1-for3 for3::*kan^R^ *ade6*-M21X *leu1*-32 *ura4*-D18 h- | This study |
| KGY13637 | *tea1*-V5_3_:hyg^R^ *ade6*-M210 *leu1*-32 *ura4*-D18 h- | This study |
| KGY13638 | *for3*-V5_3_:hyg^R^ *ade6*-M210 *leu1*-32 *ura4*-D18 h- | This study |
| KGY13864 | *tea1::tea1-for3-*V5_3_:kan^R^ *for3::*kan^R^ *ade6*-M21X *leu1*-32 *ura4*-D18 h- | This study |
| **Figure 8** | | |
| KGY246 | *ade6-*M210 *leu1-*32 *ura4-*D18 h- | Lab stock |
| KGY6008 | *fic1::ura4^+^ ade6-*M21X *leu1-*32 *ura4-*D18 h+ | Lab stock |
| KGY13592 | *tea1::tea1-for3 for3::*kan^R^ *ade6*-M21X *leu1*-32 *ura4*-D18 h- | This study |
| KGY13643 | *fic1::ura4^+^ tea1::tea1-for3 for3::*kan^R^ *ade6*-M21X *leu1*-32 *ura4*-D18 h- | This study |
| **Figure 9** | | |
| KGY246 | *ade6*-M210 *leu1*-32 *ura4*-D18 h- | Lab stock |
| KGY954 | *asp1::ura4^+^ ade6*-M21X *leu1*-32 *ura4*-D18 h- | Lab stock |
| KGY3354 | *imp2::ura4^+^ ade6*-M216 *leu1*-32 *ura4*-D18 h+ | S. Sazer |
| KGY4320 | *spn1::ura4^+^ ade6-*M21X *leu1*-32 *ura4*-D18 h+ | Lab stock |
| KGY6008 | *fic1::ura4^+^ ade6-*M21X *leu1-*32 *ura4-*D18 h+ | Lab stock |
| KGY6628 | *cdc7*-24 *ade6*-M21X *leu1*-32 *ura4*-D18 h+ | Lab stock |
| KGY6658 | *rlc1::ura4^+^ ade6*-M21X *leu1*-32 *ura4*-D18 h- | Lab stock |
| KGY7143 | *cyk3::ura4^+^* *ade6*-M21X *leu1*-32 *ura4*-D18 h+ | Lab stock |
| KGY8214 | *pxl1::*kan^R^ *ade6*-M21X *leu1*-32 *ura4*-D18 h+ | Lab stock |
| KGY12872 | *vps24::ura4^+^ ade6-*M21X *leu1*-32 *ura4-*D18 h- | K. Takegawa |
| KGY13440 | *asp1::ura4^+^ vps24::ura4^+^ ade6-*M21X *leu1*-32 *ura4-*D18 h- | This study |
| KGY13470 | *asp1::ura4^+^ fic1::ura4^+^ ade6-*M21X *leu1-*32 *ura4-*D18 h+ | This study |
| KGY13471 | *asp1::ura4^+^ spn1::ura4^+^ ade6-*M21X *leu1*-32 *ura4*-D18 h- | This study |
| KGY13592 | *tea1::tea1-for3 for3::*kan^R^ *ade6*-M21X *leu1*-32 *ura4*-D18 h- | This study |
| KGY13643 | *fic1::ura4^+^ tea1::tea1-for3 for3::*kan^R^ *ade6*-M21X *leu1*-32 *ura4*-D18 h- | This study |
| KGY14088 | *asp1::ura4^+^ pxl1::*kan^R^ *ade6-*M21X *leu1-*32 *ura4-*D18 h+ | This study |
| KGY14089 | *asp1::ura4^+^ rlc1::ura4^+^ ade6-*M21X *leu1-*32 *ura4-*D18 h- | This study |
| **Figure S1** | | |
| KGY246 | *ade6*-M210 *leu1*-32 *ura4*-D18 h- | Lab stock |
| KGY4967 | *rga1::ura4^+^* *ade6*-M21X *leu1*-32 *ura4*-D18 h- | Lab stock |
| KGY5004 | *tea1*-GFP:kan^R^ *ade6*-M21X *leu1*-32 *ura4*-D18 h- | P. Nurse |
| KGY5385 | *rgf1::ura4^+^* *ade6*-M21X *leu1*-32 *ura4*-D18 h+ | Lab stock |
| KGY6008 | *fic1::ura4^+^* *ade6*-M21X *leu1*-32 *ura4*-D18 h+ | Lab stock |
| KGY7264 | *wsp1::ura4^+^ ade6*-M21X *leu1*-32 *ura4*-D18 h- | Lab stock |
| KGY10633 | *fic1::ura4^+^ rgf1*-GFP:kan^R^ *ade6*-M21X *leu1*-32 *ura4*-D18 h+ | This study |
| KGY10774 | *fic1::ura4^+^* *tea1*-GFP:kan^R^ *ade6*-M21X *leu1*-32 *ura4*-D18 h- | This study |
| KGY10941 | *rgf1*-GFP:kan^R^ *crn1*-RFP:hyg^R^ *ade6*-M210 *leu1*-32 *ura4*-D18 h- | This study |
| KGY10942 | *fic1::ura4^+^* *rgf1*-GFP:kan^R^ *crn1*-RFP:hyg^R^ *ade6*-M21X *leu1*-32 *ura4*-D18 h- | This study |
| KGY11056 | *fic1::ura4^+^* *rgf1::ura4^+^* *ade6*-M21X *leu1*-32 *ura4*-D18 h+ | This study |
| KGY11098 | *fic1::ura4^+^* *rga1::ura4^+^* *ade6*-M21X *leu1*-32 *ura4*-D18 h- | This study |
| KGY11144 | *fic1::ura4^+^* *wsp1::ura4^+^* *ade6*-M21X *leu1*-32 *ura4*-D18 h+ | This study |
| KGY11312 | *fic1::ura4^+^* *tea4*-GFP:kan^R^ *ade6*-M21X *leu1*-32 *ura4*-D18 h- | This study |
| KGY11391 | *tea4*-GFP:kan^R^ *ade6*-M21X *leu1*-32 *ura4*-D18 h- | This study |
|  | **Figure S2** |  |
| KGY246 | *ade6*-M210 *leu1*-32 *ura4*-D18 h- | Lab stock |
| KGY11242 | *fic1N-*GFP:kan^R^ *ade6*-M210 *leu1*-32 *ura4*-D18 h- | This study |
| KGY11313 | *fic1C*-GFP:kan^R^ *ade6*-M21X *leu1*-32 *ura4*-D18 h+ | This study |
| KGY12658 | *fic1*-GFP:kan^R^ *ade6*-M210 *leu1*-32 *ura4*-D18 h- | This study |
|  | **Figure S3** |  |
| KGY11309 | *fic1*-GFP:kan^R^ *cdc15*-mCherry:kan^R^ ade6-M21X *leu1*-32 *ura4*-D18 h- | This study |
| KGY11878 | *fic1-P257A*-GFP:kan^R^ *ade6*-M21X *leu1*-32 *ura4*-D18 h+ | This study |
| KGY13834 | *fic1-P257A*-GFP:kanR *imp2::ura4^+^ cyk3::ura4^+^* *ade6*-M21X *leu1*-32 *ura4*-D18 h+ | This study |
| KGY14090 | *imp2::ura4^+^ crn1*-GFP:kan^R^ ade6-M21X *leu1*-32 *ura4*-D18 h+ | This study |
| KGY14093 | *cdc15∆SH3* *crn1*-GFP:kan^R^ *ade6*-M21X *leu1*-32 *ura4*-D18 h+ | This study |
| **Figure S4** | | |
| KGY702 | *cdc16-*116 *ade6*-M210 *leu1*-32 *ura4*-D18 h+ | Lab stock |
| KGY1105 | *sid2*-250 *ade6*-M21X *leu1*-32 *ura4*-D18 h- | Lab stock |
| KGY1287 | *spg1*-106 *ade6*-M210 *leu1*-32 *ura4*-D18 h- | Lab stock |
| KGY4387 | *eng1*-GFP:kan^R^ *ade6*-M210 *leu1*-32 *ura4*-D18 h- | Lab stock |
| KGY6008 | *fic1::ura4^+^* *ade6*-M21X *leu1*-32 *ura4*-D18 h+ | Lab stock |
| KGY6628 | *cdc7*-24 *ade6*-M21X *leu1*-32 *ura4*-D18 h+ | Lab stock |
| KGY7143 | *cyk3::ura4^+^* *ade6*-M21X *leu1*-32 *ura4*-D18 h+ | Lab stock |
| KGY10562 | *cyk3::ura4^+^ cdc7*-24 *ade6*-M21X *leu1*-32 *ura4*-D18 h+ | This study |
| KGY10564 | *cyk3::ura4^+^ cdc16*-116 *ade6*-M21X *leu1*-32 *ura4*-D18 h- | This study |
| KGY10569 | *fic1::ura4^+^ cdc16*-116 *ade6*-M21X *leu1*-32 *ura4*-D18 h+ | This study |
| KGY10578 | *fic1::ura4^+^ cdc7*-24 *ade6*-M21X *leu1*-32 *ura4*-D18 h- | This study |
| KGY10585 | *cyk3::ura4^+^ sid2*-250 *leu1*-32 *ura4*-D18 h+ | This study |
| KGY10630 | *cyk3::ura4^+^ spg1*-106 *ade6*-M21X *leu1*-32 *ura4*-D18 h- | This study |
| KGY10780 | *fic1::ura4^+^* *cdc25*-22 *ade6*-M21X *leu1*-32 *ura4*-D18 h- | This study |
| KGY13395 | *fic1::ura4^+^* *eng1*-GFP:kan^R^ *ade6*-M21X *leu1*-32 *ura4*-D18 h+ | This study |
| KGY14092 | *fic1::ura4^+^* *rlc1*-mCherry_3_:kan^R^ *ade6*-M21X *leu1*-32 *ura4*-D18 h- | This study |
| **Figure S5** | | |
| KGY246 | *ade6*-M210 *leu1*-32 *ura4*-D18 h- | Lab stock |
| KGY925 | *spn4::ura4^+^ ade6-*M21X *leu1*-32 *ura4­*-D18 h- | Lab stock |
| KGY1002 | *cdc12*-112 *ade6*-M216 *leu1*-32 *ura4*-D18 h+ | Lab stock |
| KGY1105 | *sid2*-250 *ade6*-M21X *leu1*-32 *ura4*-D18 h- | Lab stock |
| KGY1287 | *spg1*-106 *ade6*-M210 *leu1*-32 *ura4*-D18 h- | Lab stock |
| KGY1546 | *agn1::ura4^+^ade6-*M21X *leu1*-32 *ura4*-D18 h+ | Lab stock |
| KGY2153 | *cps1*-191 *ade6*-M210 *ura4*-D18 *lys1*-131 h- | Lab stock |
| KGY2971 | *myo2*-E1 *ade6*-M216 *leu1*-32 *ura4*-D18 h+ | M. Balasubramanian |
| KGY3135 | *mid2::ura4^+^ade6*-M21X *leu1*-32 *ura4*-D18 h- | Lab stock |
| KGY3354 | *imp2::ura4^+^ ade6*-M216 *leu1*-32 *ura4*-D18 h+ | S. Sazer |
| KGY4320 | *spn1::ura4^+^ ade6-*M21X *leu1*-32 *ura4*-D18 h+ | Lab stock |
| KGY5032 | *eng1::*kan^R^ *ura4*-D18 h- | Lab stock |
| KGY6628 | *cdc7*-24 *ade6*-M21X *leu1*-32 *ura4*-D18 h+ | Lab stock |
| KGY6658 | *rlc1::ura4^+^ ade6*-M21X *leu1*-32 *ura4*-D18 h- | Lab stock |
| KGY7870 | *sst2::ura4^+^ ade6*-M210 *leu1*-32 *ura4*-D18 h- | Lab stock |
| KGY8214 | *pxl1::*kan^R^ *ade6*-M21X *leu1*-32 *ura4*-D18 h+ | Lab stock |
| KGY12869 | *vps2::LEU2^+^* *leu1-*32 *ura4-*C190T h- | K. Takegawa |
| KGY12872 | *vps24::ura4^+^ ade6-*M21X *leu1*-32 *ura4-*D18 h- | K. Takegawa |
| KGY13546 | *vps24::ura4^+^ sid2*-250 *ade6-*M21X *leu1*-32 *ura4-*D18 h- | This study |
| KGY13551 | *vps24::ura4^+^ myo2*-E1 *ade6-*M21X *leu1*-32 *ura4-*D18 h- | This study |
| KGY13552 | *vps24::ura4^+^ cdc12*-112 *ade6-*M21X *leu1*-32 *ura4-*D18 h- | This study |
| KGY13554 | *sst2::ura4^+^ myo2*-E1 *ade6-*M21X *leu1*-32 *ura4-*D18 h- | This study |
| KGY13558 | *sst2::ura4^+^ spg1*-106 *ade6-*M21X *leu1*-32 *ura4-*D18 h+ | This study |
| KGY13561 | *sst2::ura4^+^ cdc12*-112 *ade6-*M21X *leu1*-32 *ura4-*D18 h+ | This study |
| KGY13564 | *vps2::LEU2^+^* *myo2*-E1 *ade6*-M21X *leu1*-32 *ura4^-^* h+ | This study |
| KGY13567 | *vps2::LEU2^+^* *spg1*-106 *ade6*-M21X *leu1*-32 *ura4^-^* h- | This study |
| KGY13570 | *vps2::LEU2^+^* *cps1*-191 *ade6*-M21X *leu1*-32 *ura4^-^* h+ | This study |
| KGY13572 | *vps2::LEU2^+^* *imp2::ura4^+^ leu1*-32 *ura4^-^* h- | This study |
| KGY13573 | *vps2::LEU2^+^* *sid2*-250 *ade6*-M21X *leu1*-32 *ura4^-^* h+ | This study |
| KGY13574 | *sst2::ura4^+^ cps1*-191 *ade6*-M21X *leu1*-32 *ura4-*D18 *lys1*-131 h+ | This study |
| KGY13575 | *sst2::ura4^+^ imp2::ura4^+^ ade6-*M21X *leu1*-32 *ura4-*D18 h+ | This study |
| KGY13589 | *vps24::ura4^+^ imp2::ura4^+^ ade6-*M21X *leu1*-32 *ura4-*D18 h+ | This study |
|  | **Figure S6** |  |
| KGY2540 | *cdc10-*V50 *ade6-*M210 *leu1*-32 *ura4*-D18 h+ | Lab stock |
| KGY13678 | *cdc10*-V50 *tea1::tea1-for3 for3::*kan^R^ *ade6*-M21X *leu1*-32 *ura4*-D18 h-­ | This study |
|  | **Figure S7** |  |
| KGY246 | *ade6*-M210 *leu1*-32 *ura4*-D18 h- | Lab stock |
| KGY3354 | *imp2::ura4^+^ ade6*-M216 *leu1*-32 *ura4*-D18 h+ | S. Sazer |
| KGY4320 | *spn1::ura4^+^ ade6-*M21X *leu1*-32 *ura4*-D18 h+ | Lab stock |
| KGY6008 | *fic1::ura4^+^* *ade6*-M21X *leu1*-32 *ura4*-D18 h+ | Lab stock |
| KGY6628 | *cdc7*-24 *ade6*-M21X *leu1*-32 *ura4*-D18 h+ | Lab stock |
| KGY7143 | *cyk3::ura4^+^* *ade6*-M21X *leu1*-32 *ura4*-D18 h+ | Lab stock |
| KGY9065 | *tea1::ura4^+^ leu1*-32 *ura4*-D18 h- | P. Nurse |
| KGY12872 | *vps24::ura4^+^ ade6-*M21X *leu1*-32 *ura4-*D18 h- | K. Takegawa |
| KGY13643 | *fic1::ura4^+^ tea1::tea1-for3 for3::*kan^R^ *ade6*-M21X *leu1*-32 *ura4*-D18 h- | This study |
